# Clinical aspects and the quality of life among women with endometriosis and infertility: a cross-sectional study

**DOI:** 10.1186/s12905-020-00987-7

**Published:** 2020-06-12

**Authors:** Marina Pessoa de Farias Rodrigues, Fabia Lima Vilarino, Alessandra de Souza Barbeiro Munhoz, Laércio da Silva Paiva, Luiz Vinicius de Alcantara Sousa, Victor Zaia, Caio Parente Barbosa

**Affiliations:** 1 Faculdade de Medicina do ABC / Centro Universitário Saúde ABC, Avenida Lauro Gomes, 2000, Vila Sacadura Cabral, Santo André, SP 09060-870 Brazil; 2 Centro Universitário Vale do Salgado, Icó, Ceará Brazil; 3 Instituto Ideia Fértil de Saúde Reprodutiva, Santo André, SP Brazil; 4 Laboratório de Epidemiologia e Análises de Dados da Faculdade de Medicina do ABC / Centro Universitário Saúde ABC, Santo André, SP Brazil; 5 Disciplina de Saúde Sexual, Reprodutiva e Genética e Pós-Graduação em Ciências da Saúde da Faculdade de Medicina do ABC / Centro Universitário Saúde ABC, Santo André, SP Brazil

**Keywords:** Endometriosis, Quality of life, Infertility

## Abstract

**Background:**

The quality of life (QoL) of patients with endometriosis and infertility was assessed in different stages and correlated with the clinical features of the cases.

**Methods:**

The present study was a cross-sectional study; 106 women were included, divided in two endometriosis groups (Grade I/II, 26 women, and Grade II/IV, 74 women). All participants attended the Endometriosis and Infertility Outpatient Clinic of the Instituto Ideia Fértil de Saúde Reprodutiva, Faculdade de Medicina do ABC, São Paulo, Brazil, were and responded to the Short Form (SF) Health Survey-36. Convenience sampling was used due to the authors’ access to the study population; however, the sample number was calculated to be sufficient for 95% power in both groups.

**Results:**

Homogeneity was observed between Grade I/II and Grade III/IV staging, with similar mean ages (35.27, ±3.64 years and 34.04, ±3.39 years, respectively, *p* = 0.133); types of infertility (*p* = 0.535); infertility time (*p* = 0.654); degrees of pain (*p* = 0.849); and symptoms common to endometriosis, namely, dysmenorrhea (*p* = 0.841), dyspareunia (0.466), chronic pelvic pain (*p* = 0.295), and intestinal (*p* = 0.573) or urinary (*p* = 0.809) diseases. Comparisons of median scores in the QoL domains demonstrated that the distributions of QoL and clinical symptoms were significantly related between the types of dyspareunia and the following domains: physical functioning (*p* = 0.017), role- emotional (*p* = 0.013), and general health (*p* = 0.001). Regarding pain outside of menstruation, there was significance in the pain domain (*p* = 0.017), and degree of pain was significance in physical functioning (*p* = 0.005) and role-physical (*p* = 0.011) domains.

**Conclusions:**

The present study pointed out that it is not the stage of endometriosis that interferes in the quality of life of women with endometriosis and infertility but rather the clinical manifestations, such as dyspareunia and pain. Thus, we can conclude that the patient’s perception of the disease should be considered in health care and that the losses are independent of the degree of endometriosis in this population with the aggravating factor of infertility.

## Background

Endometriosis is a heterogeneous disease characterized by the presence of endometrial tissue outside the uterine cavity. It may be asymptomatic or could include clinical manifestations such as chronic pelvic pain, dysmenorrhea, dyspareunia, dysuria, pain after the menstrual period, and infertility [1–4].

Endometriosis occurs in women in the reproductive phase with a high incidence, and worsens their quality of life (QoL) [5, 6], causing discomfort, psychic, physiological, marital, and social liability [7].

It is a disease that can lead to social isolation; and such behavior may be related to pain and fatigue that also trigger psychological alterations; loss of productivity and yield at work; whilst its recurrence has the greatest negative impact on psychological health, vitality, financial conditions, and reduction in social activities [8–11].

According to recent data, endometriosis is a very complex condition, and psychological aspects play an important role in determining both, its severity of symptoms and effectiveness of treatments [12].

Due to the chronicity of endometriosis, it may be associated with considerable physical and emotional morbidity; and it is also known that disease carriers experience harm in their daily activities, which has an economic impact due to a reduction or loss of working hours and hospitalizations [13, 14].

The reduction of QoL in this population can be explained by the complexity of disease etiology and manifestations, as well as by the interference in women’s reproductive capacity. In addition, treatment does not necessarily guarantee a cure or complete remission of symptoms but may only contribute to improving the patients’ QoL [15].

Since endometriosis is one of the most common benign gynecological diseases, it has a 10% prevalence among women of reproductive age [16]. Patients with endometriosis are 20 times more likely to experience infertility; in addition to its being considered a cause for spontaneous abortion [17]. Moreover, 25–50% of infertile women have endometriosis, and 30–50% of women with endometriosis are infertile [18].

The present study’s aim was to verify the levels of QoL in women with endometriosis and infertility; and to compare these levels between staging groups as well as the clinical symptoms of endometriosis with aggravating factors of infertility. This proposal would be of interest in providing improvements and specificity in the monitoring of this population, considering interdisciplinary aspects.

## Methods

### Design and setting of the study

For verifying QoL by comparing the staging groups and clinical symptoms of endometriosis, this cross-sectional and observational study that was carried out at the Instituto Ideia Fértil (IIF), Faculdade de Medicina do ABC, Santo André - SP, Brazil used a quantitative approach and adopted the STROBE guidelines [19].

### Characteristics of participants and setting

A total of 106 women, who did not become pregnant 6 months after diagnosis by laparoscopy; and who had attended the IIF Endometriosis and Infertility Clinic from April to December 2015, were included. They were recruited after a specific consultation in the endometriosis outpatient clinic and were personally invited by the first author. From the 210 who were invited, 106 accepted; while those who declined reported no interest in research.

A convenience sample was used because the authors had direct access to the IIF endometriosis outpatient clinic, and the minimum sample size of 100 participants was stipulated by a free statistical software program (G*Power Software) to achieve 95% power considering the proposed analytical model, studied variables, and the number of groups.

Considering the differences in symptomatology described in the literature; the participants were divided according to the staging of endometriosis into two groups: stage I/II (26 women) and stage III/IV (80 women).

All the patients had only undergone a laparoscopy, in which the diagnosis of endometriosis was made. They had performed the laparoscopy procedure due to their symptom of chronic pain.

### Selection criteria

The inclusion criteria were infertile and endometriosis women whose diagnosis and staging of the disease had been confirmed by laparoscopy; and those who had agreed to participate in the study and had signed the consent and post-consent forms. The exclusion criteria comprised: age less than 18 years; carriers of endometriosis whose infertility included an associated male factor; used analgesics or anti-inflammatory drugs or hormonal treatment in the past 3 months; as well as diagnosed and/or being treated for depression or anxiety; that were all factors that could interfere with responses to the Medical Outcomes Study 36-item short-form health survey (SF-36) questionnaire.

### Data collection and measures

The participants were personally invited to participate in the research, only if they accepted, signed the informed consent form, responded to the SF-36, and gave researchers access to their medical records. Each participant took, on an average, 15 min to complete the questionnaire.

For data collection, the SF-36 [20], which measures impairment in an individual’s QoL in a generic manner was used. Since it was validated in 1999 in Brazil by Ciconelli et al. [21], as well as in 2014, for the population with endometriosis [22]; it facilitates measuring the QoL of patients with endometriosis, and can be used as a prognostic indicator of clinical improvement [23]. The SF-36 which evaluates eight QoL domains: physical functioning, role-physical, bodily pain, role-emotional, general health, vitality, social functioning, and mental health; is among the most used instruments worldwide [14, 15, 22–29]. The cutoff points or domains for interpreting the QoL levels were based on the Bieleman study [30], which adopted a criterion in which values above 60 points (on a scale of 0 to 100) indicated preservation of QoL.

The clinical data verified in the electronic medical record included age; infertility time (in years); menarche age; infertility type (primary or secondary); previous oral conceptive pill use (yes-no) and usage time (in years); miscarriage (yes-no); staging of endometriosis according to the “Revised American Society for Reproductive Medicine (ASRM) classification of endometriosis: 1996” [31]; and confirmation of endometriosis from the results of a pathology examination. The degree of pain during menstruation was assessed by a clinical questionnaire on five levels: 0-absent, 1-mild, 2-moderate, 3-severe, and 4-disabling. The presence or absence of dyspareunia was assessed as: superficial – pain in entrance of the vagina, deeper – pain during penetration or thrusting of the penis, and superficial and deeper – both types) [32]. Similarly, chronic pelvic pain, dysmenorrhea, intestinal (tenesmus and/or enterorrhagia during menstruation), and urinary (dysuria and/or hematuria during menstruation) disorders were also assessed. All medical visits were performed by a gynecologist specialized in endometriosis and infertility (the third author).

### Statistics

The descriptive variables were verified using frequency analysis. The non-normal quantitative variables were presented as medians and interquartile ranges (IQR), the normal quantitative variables were presented as means and Standard Deviation (SD). The power for the intragroup tests was 95% for both groups, tested by the G*Power Software. The data missing were verified and found to be non-existent. The data were verified for normality through the Kolmogorov-Smirnov test, with a partially non normal distribution; to reach the proposed goal, nonparametric and parametric tests were used. Mann-Whitney tests or t-Tests were used to verify the QoL domains’ relationship with the type of infertility; and the degree of endometriosis and QoL with the profile of the participants. Kruskal-Wallis or ANOVA tests were used to verify the association between the QoL domains and the clinical aspects of endometriosis or the participants’ profiles. The chi-squared test was also used to associate the profile of the participants with the staging of endometriosis. Spearman or Pearson’s correlations were used for continuous variables (complete analysis can access in supplementary material). The program for statistical analyses was SPSS 21 for Windows. Considering the difference in the sample size of the groups, the specific “n power” for the intergroup comparison tests was calculated to be 73% in the t-Test/Mann-Whitney test and 99% in the ANOVA/Kruskal-Wallis tests, both with a medium effect size. The significance level adopted for all analyses was *p* ≤ 0.05.

## Results

### Patient profiles

The 106 participants, who were divided according to stage I/II (*n* = 26) and stage III/IV (*n* = 80), exhibited mean ages of 35.27 ± 3.64 years and 34.04 ± 3.27 years, respectively, (*p* = 0.133). Both groups underwent laparoscopy and were homogeneous for the type of infertility (*p* = 0.536), menarche age (*p* = 0.254), infertility time (*p* = 0.654), miscarriage (*p* = 0.528), previous oral conceptive pill usage (*p* = 0.606), degree of pain (*p* = 0.194), dysmenorrhea (*p* = 0.841), dyspareunia (*p* = 0.466), chronic pelvic pain (*p* = 0.295), intestinal disorders (*p* = 0.573), and urinary disorders (*p* = 0.809). The stage III/IV group had used contraceptive pills longer than the stage I/II group (*p* = 0.012) (Table 1).

**Table 1:** Comparison of the clinical profile of participants with endometriosis and infertility according to endometriosis staging

Clinical features	Staging of endometriosis (ASRM)		p^**a**^
	Degree I/II (***n*** = 26, 24.5%)	Degree III/IV (***n*** = 80, 75.5%)	
	n (%)		
Type of Infertility			
Primary	23 (88.5)	69 (86.3)	0.535
Secondary	3 (11.5)	11 (13.7)	
Miscarriage			
Yes	20 (76.9)	66 (82.5)	0.528
No	6 (31.1)	14 (17.5)	
Previous oral conceptive pill use			
Yes	24 (92.3)	76 (95.0)	0.606
No	2 (7.7)	4 (5.0)	
Degree of pain			
Absent	5 (19.2)	12 (15.0)	0.849
Light	2 (7.7)	6 (7.5)	
Moderate	9 (34.6)	21 (26.3)	
Serious	8 (30.8)	33 (41.3)	
Incapacitating	2 (7.7)	8 (10.0)	
Dysmenorrhea			
Absent	4 (15.4)	16 (20.0)	0.841
Primary	10 (38.5)	27 (33.8)	
Secondary	12 (46.2)	37 (43.2)	
Dyspareunia			
Absent	14 (53.8)	42 (52.5)	0.466
Deeper	4 (15.4)	9 (11.3)	
Superficial	7 (26.9)	17 (21.3)	
Deeper and Superficial	1 (3.8)	12 (15.0)	
Chronic pelvic pain			
Absent	19 (73.1)	66 (82.5)	0.295
Present	7 (26.9)	14 (17.5)	
Intestinal Disorders			
Absent	6 (23.1)	23 (28.8)	0.573
Present	20 (76.9)	57 (71.2)	
Urinary Disorders			
Absent	25 (96.2)	76 (95.0)	0.809
Present	1 (3.8)	4 (5.0)	
	**Median (IQR)**		**p**^**b**^
Infertility Time (Years)	4.50 (3.00)	4.00 (4.00)	0.654
Menarche Age (Years)	12.00 (2.00)	12.00 (2.00)	0.254
	**Mean (SD)**		**p**^**c**^
Age, mean (SD)	35.27 (3.64)	34.04 (3.27)	0.133
Oral Contraceptive pill usage time (Years)	4.80 (4.31)	7.47 (5.11)	**0.012**

*IQR* interquartile range, *SD* standard deviation. ^a^Chi-square, ^b^Mann-Whitney, ^c^Test-T

### QoL related to disease staging

No statistically significant differences were found in the QoL domains between the groups based on the degree of endometriosis. Moreover, most domains exhibited good scores. When the adopted cutoffs were verified, lower values were identified for stage I/II in the domains of general health (mean 58.69, SD ±16.56), vitality (mean 54.42, SD ±14.72), and mental health (mean 59.54, SD ±21.18); and for stage III/IV in the domains of pain (median 57.00, IQR 43.00), vitality (mean 56.24, SD ±11.38), and mental health (mean 59.23, SD ±18.52) (Table 2).

**Table 2:** Comparisons of domains of quality of life with the staging of endometriosis

Quality of life domains	Staging Endometriosis		
	Staging I/ II	Staging III/IV	p^**a**^
	Median scores (IQR)		
Physical Functioning	87.50 (25.00)	85.00 (28.00)	0.708
Role-Physical	100.00 (100.00)	100.00 (69.00)	0.794
Bodily Pain	73.00 (43.00)	**57.00 (43.00)**	0.352
Role-Emotional	66.67 (100.00)	66.67 (66.67)	0.360
	**Mean scores (SD)**		**p**^**b**^
General Health	**58.69 (16.56)**	60.54 (17.57)	0.629
Vitality	**54.42 (14.72)**	**56.24 (11.38)**	0.569
Social Functioning	66.34 (26.40)	66.20 (23.58)	0.980
Mental Health	**59.54 (21.18)**	**59.23 (18.52)**	0.946

^a^Mann-Whitney. ^b^T-Test. SD: standard deviation. *IQR* interquartile range

### QoL and clinical symptomatology of endometriosis

After considering the homogeneity of the clinical characteristics of endometriosis between the groups studied, that may have been due to the laparoscopy performed 6 months prior; it was decided to verify possible associations between the clinical characteristics of endometriosis and the QoL of the patients, without any distinctions between the mentioned groups. The scores of the QoL domains of the SF-36 were compared with all the clinical symptom types and profiles of participants. The results showed that the distribution of QoL differed significantly between the types of dyspareunia and the following domains: general health (*p* = 0.001), role-emotional (*p* = 0.013) and physical functioning (*p* = 0.017); with the “penetration” group presenting the lowest value between the previous oral conceptive pill use and role-emotional (*p* = 0.020); between the chronic pelvic pain and bodily pain (*p* = 0.017); and between degree of pain and physical functioning (*p* = 0.005); and role-physical (*p* = 0.011). The domains of the SF-36 that exhibited some significant differences by clinical symptomatology are described in Table 3.

**Table 3:** Only statistically significant comparisons between the scores of the quality of life domains evaluated by the SF36 and clinical symptomatology

Features	General Health	p	Role-Emotional	p	Physical Functioning	p	Role-Physical	p	Bodily Pain	p
	Mean (SD)		Median (IQR)		Median (IQR)		Median (IQR)		Median (IQR)	
Dyspareunia										
Absent	65.37 (16.16)	0.001^a^	83.33 (66.67)	0.013^b^	92.50 (20.00)	0.017^b^				
Deeper	48.39 (13.16)		33.34 (100.00)		75.00 (33.00)					
Superficial	59.54 (16.53)		100.00 (58.75)		87.50 (31.00)					
Deeper and Superficial	50.00 (18.10)		33.34 (83.34)		80.00 (28.00)					
Previous oral conceptive pill use										
Yes			67.00 (66.67)	0.020^c^						
No			0.00 (50.00)							
Degree of pain										
Absent					95.00 (10.00)	0.005^b^	100.00 (0.00)	0.011^b^		
Light					62.50 (36.00)		37.50 (94.00)			
Moderate					87.50 (25.00)		100.00 (50.00)			
Serious					85.00 (25.00)		75.00 (88.00)			
Incapacitating					85.00 (25.00)		100.00 (34.00)			
Chronic pelvic pain										
Absent									52.00 (43.00)	0.017^c^
Present									84.00 (30.00)	

^a^ANOVA, ^b^Kruskal-Wallis, ^c^Mann-Whitney. *SD* standard deviation, *IQR* interquartile range

## Discussion

From the results, it can be verified that the staging of endometriosis in the present sample was not associated with a difference in their QoL scores. This suggests that the reduction of QoL in the infertile and endometriosis population would require a more complex explanation than just the stages of endometriosis [15, 33]; and that infertility associated with endometriosis would impair QoL [34].

In this sense, the association of QoL with clinical manifestations rather than the degree of endometriosis as observed in this study, may be partially justified due to the homogeneity of the groups’ characteristics due to the laparoscopy that had been previously performed. Moreover, since the pain related to endometriosis was not explained by the disease itself [35], this suggests aspects related to the clinical manifestation with subsequent QoL impairment. Such an association can be verified in dyspareunia and degree of pain, which tend to interfere with the activities of daily living, causing, for example, mood swings and pain [36].

The physiological aspect should, therefore, be considered. An Italian study [37] identified that treatment for endometriosis reduced pain symptoms, such as dysmenorrhea, dyspareunia, and dysuria; and the reduction was positively associated with QoL. Thus, the pain sensations that impaired the perception of health can be seen in the present study in the relationship between degrees of pain and dyspareunia as well as the QoL domains (physical functioning, role-physical, role-emotional and general health).

Dyspareunia was the clinical symptom that was most associated with lower levels of QoL in the present study, and this finding was also corroborated by Caruso et al. [26]. Although dyspareunia is generally associated with more advanced degrees of the disease; women with minimal pelvic involvement may also experience intense pain, which again supports that the discomfort of the clinical manifestations of endometriosis does not occur exclusively because of staging [12, 38]. Therefore, physical and mental aspects may interfere with QoL [27].

The present study found QoL levels that were below the cutoff values in the following domains: vitality, general health, pain, and mental health. These results are consistent with studies from other countries, such as Austria [39] and Sweden [40], which used the SF-36, and the literature review of Silva and Marqui [29]. The reduced scores in these domains deserve attention because it indicates that individuals tended to feel tired most of the time; evaluated their personal health as precarious; experienced pain that was severe and limiting; and felt the presence of a constant feeling of nervousness, anxiety, stress, and depression [41, 42].

Finally, the present research was carried out in a reference center that specialized in endometriosis and infertility, which explains the high number of women with grade III/IV (75.5%). The present study’s generalizations are limited by aspects such as the numerical differences between the groups of endometriosis that were compared; limited number of participants in the subgroups of symptoms of endometriosis and profiles of the participants; the use of a single reference center to perform the research characterizing a convenience sample; not investigating coexisting autoimmune disease; and studying a population with endometriosis and infertility. However, this study has the following strengths: a precise examination of the population with endometriosis and infertility; confirmation of endometriosis staging by laparoscopy; the use of an internationally validated scale for QoL; and electronic medical records collected by a gynecologist specialized in endometriosis and infertility (third author).

## Conclusion

The present study demonstrated that the clinical manifestations of endometriosis such as dyspareunia and pain, interfered with the QoL levels, whereas the stages of endometriosis did not interfere. These findings indicate that the participants’ perception of endometriosis and infertility are aspects that should be considered in health care, since the loss of QoL would not depend directly on the staging of the disease but on how the participants perceive it.

## Supplementary information

**Additional file 1.**

## Data Availability

The all datasets used and/or analyzed during the current study are available from the corresponding author on reasonable request.

## Abbreviations

ANOVA: Analysis of variance
ASRM: American Society of Reproductive Medicine
IFF: Instituto Ideia Fértil
IQR: Interquartile Ranges
QoL: Quality of Life
SD: Standard Deviation
SF-36: Short-Form Health Survey
SPSS: Statistical Package for the Social Sciences
STROBE: Strengthening the Reporting of Observational studies in Epidemiology
