# Supplementary material for: Clinical aspects and the quality of life among women with endometriosis and infertility: a cross-sectional study
Source: BMC Womens Health. 2020 Jun 12;20:124. doi: 10.1186/s12905-020-00987-7 (PMC7291762; doi:10.1186/s12905-020-00987-7)
Supplement: Supplementary file 1 — Additional file 1. [file 12905_2020_987_MOESM1_ESM.pdf]

```
 *Nonparametric Tests: One Sample.
NPTESTS
  /ONESAMPLE TEST (idade temp_infert menarca tempo_ACO cap_func lim_fisico dor estado_geral vit
  /MISSING SCOPE=ANALYSIS USERMISSING=EXCLUDE
  /CRITERIA ALPHA=0.05 CILEVEL=95.
```

# Nonparametric Tests

**Notes**

| | | |
|---|---|---|
| Output Created | | 27-APR-2020 16:05:21 |
| Comments | | |
| Input | Data | \\Mac\iCloud\Lavoro\FMABC\Orientações\Orientações\finite\Marina Rodrigues - Caio e Fabia\Marina Farias\data_article_4.sav |
| | Active Dataset | InsiemeDati1 |
| | Filter | <none> |
| | Weight | <none> |
| | Split File | <none> |
| | N of Rows in Working Data File | 106 |
| Syntax | | NPTESTS<br>  /ONESAMPLE TEST (idade temp_infert menarca tempo_ACO cap_func lim_fisico dor estado_geral vitalidade asp_sociais lim_emocional saude_mental)<br>  /MISSING SCOPE=ANALYSIS USERMISSING=EXCLUDE<br>  /CRITERIA ALPHA=0.05 CILEVEL=95. |
| Resources | Processor Time | 00:00:00,27 |
| | Elapsed Time | 00:00:00,17 |

```
[InsiemeDati1] \\Mac\iCloud\Lavoro\FMABC\Orientações\Orientações\finite\Marin
a Rodrigues - Caio e Fabia\Marina Farias\data_article_4.sav
```

## Hypothesis Test Summary

| | Null Hypothesis | Test | Sig. | Decision |
|---|---|---|---|---|
| **1** | The distribution of idade(anos) is normal with mean 34,340 and standard deviation 3,39. | One-Sample Kolmogorov-Smirnov Test | ,092 | Retain the null hypothesis. |
| **2** | The distribution of Tempo de infertilidade em anos is normal with mean 4,453 and standard deviation 2,67. | One-Sample Kolmogorov-Smirnov Test | ,040 | Reject the null hypothesis. |
| **3** | The distribution of idade menarca is normal with mean 12,311 and standard deviation 1,39. | One-Sample Kolmogorov-Smirnov Test | ,023 | Reject the null hypothesis. |
| **4** | The distribution of tempo de uso de AC is normal with mean 6,820 and standard deviation 5,04. | One-Sample Kolmogorov-Smirnov Test | ,085 | Retain the null hypothesis. |
| **5** | The distribution of SF-36 - capacidade funcional is normal with mean 81,991 and standard deviation 18,95. | One-Sample Kolmogorov-Smirnov Test | ,004 | Reject the null hypothesis. |
| **6** | The distribution of SF-36 - limitacao fisica is normal with mean 71,972 and standard deviation 38,80. | One-Sample Kolmogorov-Smirnov Test | ,000 | Reject the null hypothesis. |
| **7** | The distribution of SF-36 - dor is normal with mean 62,472 and standard deviation 25,40. | One-Sample Kolmogorov-Smirnov Test | ,002 | Reject the null hypothesis. |
| **8** | The distribution of SF-36 - estado geral is normal with mean 60,085 and standard deviation 17,27. | One-Sample Kolmogorov-Smirnov Test | ,248 | Retain the null hypothesis. |

Asymptotic significances are displayed. The significance level is ,05.

(continued)

### Hypothesis Test Summary

| | Null Hypothesis | Test | Sig. | Decision |
|---|---|---|---|---|
| **9** | The distribution of SF-36 - vitalidade is normal with mean 55,792 and standard deviation 12,23. | One-Sample Kolmogorov-Smirnov Test | ,080 | Retain the null hypothesis. |
| **10** | The distribution of SF-36 - aspectos sociais is normal with mean 66,236 and standard deviation 24,17. | One-Sample Kolmogorov-Smirnov Test | ,110 | Retain the null hypothesis. |
| **11** | The distribution of SF-36 - limites emocionais is normal with mean 62,865 and standard deviation 40,71. | One-Sample Kolmogorov-Smirnov Test | ,000 | Reject the null hypothesis. |
| **12** | The distribution of SF-36 - saúde mental is normal with mean 59,302 and standard deviation 19,10. | One-Sample Kolmogorov-Smirnov Test | ,331 | Retain the null hypothesis. |

Asymptotic significances are displayed. The significance level is ,05.

```
 SORT CASES  BY Grau_Endometriodo_Cat.
SPLIT FILE LAYERED BY Grau_Endometriodo_Cat.
EXAMINE VARIABLES=idade temp_infert menarca tempo_ACO
  /PLOT NONE
  /STATISTICS DESCRIPTIVES
  /CINTERVAL 95
  /MISSING LISTWISE
  /NOTOTAL.
```

# Explore

**Notes**

| | | |
|---|---|---|
| Output Created | | 08-MAY-2020 09:13:32 |
| Comments | | |
| Input | Data | \\Mac\iCloud\Lavoro\FMABC\Orientações\Orientações\finite\Marina Rodrigues - Caio e Fabia\Marina Farias\data_article_4.sav |
| | Active Dataset | InsiemeDati1 |
| | Filter | <none> |
| | Weight | <none> |
| | Split File | Grau_Endometriodo_Cat |
| | N of Rows in Working Data File | 106 |
| Missing Value Handling | Definition of Missing | User-defined missing values for dependent variables are treated as missing. |
| | Cases Used | Statistics are based on cases with no missing values for any dependent variable or factor used. |
| Syntax | | EXAMINE VARIABLES=idade temp_infert menarca tempo_ACO /PLOT NONE /STATISTICS DESCRIPTIVES /CINTERVAL 95 /MISSING LISTWISE /NOTOTAL. |
| Resources | Processor Time | 00:00:00,02 |
| | Elapsed Time | 00:00:00,02 |

```
[InsiemeDati1] \\Mac\iCloud\Lavoro\FMABC\Orientações\Orientações\finite\Marin
a Rodrigues - Caio e Fabia\Marina Farias\data_article_4.sav
```

**Case Processing Summary**

| | | Cases | | | | | |
|---|---|---|---|---|---|---|---|
| | | Valid | | Missing | | Total | |
| Grau_Endometriodo_Cat | | N | Percent | N | Percent | N | Percent |
| Grau I/II | idade | 26 | 100,0% | 0 | 0,0% | 26 | 100,0% |
| | temp_infert | 26 | 100,0% | 0 | 0,0% | 26 | 100,0% |
| | menarca | 26 | 100,0% | 0 | 0,0% | 26 | 100,0% |
| | tempo_ACO | 26 | 100,0% | 0 | 0,0% | 26 | 100,0% |
| Grau III/IV | idade | 80 | 100,0% | 0 | 0,0% | 80 | 100,0% |
| | temp_infert | 80 | 100,0% | 0 | 0,0% | 80 | 100,0% |
| | menarca | 80 | 100,0% | 0 | 0,0% | 80 | 100,0% |
| | tempo_ACO | 80 | 100,0% | 0 | 0,0% | 80 | 100,0% |

**Descriptives**

| Grau_Endometriodo_Cat | | | | Statistic | Std. Error |
|---|---|---|---|---|---|
| Grau I/II | idade | Mean | | 35,27 | ,714 |
| | | 95% Confidence Interval for Mean | Lower Bound | 33,80 | |
| | | | Upper Bound | 36,74 | |
| | | 5% Trimmed Mean | | 35,27 | |
| | | Median | | 35,50 | |
| | | Variance | | 13,245 | |
| | | Std. Deviation | | 3,639 | |
| | | Minimum | | 28 | |
| | | Maximum | | 43 | |
| | | Range | | 15 | |
| | | Interquartile Range | | 5 | |
| | | Skewness | | -,245 | ,456 |
| | | Kurtosis | | ,000 | ,887 |
| | temp_infert | Mean | | 4,54 | ,487 |
| | | 95% Confidence Interval for Mean | Lower Bound | 3,53 | |
| | | | Upper Bound | 5,54 | |
| | | 5% Trimmed Mean | | 4,41 | |
| | | Median | | 4,50 | |
| | | Variance | | 6,178 | |
| | | Std. Deviation | | 2,486 | |
| | | Minimum | | 1 | |
| | | Maximum | | 11 | |
| | | Range | | 10 | |
| | | Interquartile Range | | 3 | |
| | | Skewness | | ,543 | ,456 |
| | | Kurtosis | | ,383 | ,887 |
| | menarca | Mean | | 12,58 | ,230 |
| | | 95% Confidence Interval for Mean | Lower Bound | 12,10 | |
| | | | Upper Bound | 13,05 | |
| | | 5% Trimmed Mean | | 12,54 | |
| | | Median | | 12,00 | |
| | | Variance | | 1,374 | |
| | | Std. Deviation | | 1,172 | |
| | | Minimum | | 11 | |
| | | Maximum | | 15 | |
| | | Range | | 4 | |
| | | Interquartile Range | | 2 | |
| | | Skewness | | ,446 | ,456 |
| | | Kurtosis | | -,990 | ,887 |

**Descriptives**

| Grau_Endometriodo_Cat | | | | Statistic | Std. Error |
|---|---|---|---|---|---|
| Grau III/IV | tempo_ACO | Mean | | 4,8038 | ,84532 |
| | | 95% Confidence Interval for Mean | Lower Bound | 3,0629 | |
| | | | Upper Bound | 6,5448 | |
| | | 5% Trimmed Mean | | 4,5043 | |
| | | Median | | 4,0000 | |
| | | Variance | | 18,579 | |
| | | Std. Deviation | | 4,31030 | |
| | | Minimum | | ,00 | |
| | | Maximum | | 15,00 | |
| | | Range | | 15,00 | |
| | | Interquartile Range | | 7,00 | |
| | | Skewness | | 1,012 | ,456 |
| | | Kurtosis | | ,508 | ,887 |
| | idade | Mean | | 34,04 | ,366 |
| | | 95% Confidence Interval for Mean | Lower Bound | 33,31 | |
| | | | Upper Bound | 34,77 | |
| | | 5% Trimmed Mean | | 34,08 | |
| | | Median | | 34,50 | |
| | | Variance | | 10,720 | |
| | | Std. Deviation | | 3,274 | |
| | | Minimum | | 27 | |
| | | Maximum | | 43 | |
| | | Range | | 16 | |
| | | Interquartile Range | | 4 | |
| | | Skewness | | -,221 | ,269 |
| | | Kurtosis | | ,239 | ,532 |
| | temp_infert | Mean | | 4,43 | ,307 |
| | | 95% Confidence Interval for Mean | Lower Bound | 3,81 | |
| | | | Upper Bound | 5,04 | |
| | | 5% Trimmed Mean | | 4,19 | |
| | | Median | | 4,00 | |
| | | Variance | | 7,539 | |
| | | Std. Deviation | | 2,746 | |
| | | Minimum | | 1 | |
| | | Maximum | | 14 | |
| | | Range | | 13 | |
| | | Interquartile Range | | 4 | |
| | | Skewness | | 1,163 | ,269 |
| | | Kurtosis | | 1,898 | ,532 |

**Descriptives**

| Grau_Endometriodo_Cat | | | | Statistic | Std. Error |
|---|---|---|---|---|---|
| menarca | Mean | | | 12,23 | ,162 |
| | 95% Confidence Interval for Mean | Lower Bound | | 11,90 | |
| | | Upper Bound | | 12,55 | |
| | 5% Trimmed Mean | | | 12,19 | |
| | Median | | | 12,00 | |
| | Variance | | | 2,101 | |
| | Std. Deviation | | | 1,449 | |
| | Minimum | | | 9 | |
| | Maximum | | | 16 | |
| | Range | | | 7 | |
| | Interquartile Range | | | 2 | |
| | Skewness | | | ,209 | ,269 |
| | Kurtosis | | | ,012 | ,532 |
| tempo_ACO | Mean | | | 7,4750 | ,57119 |
| | 95% Confidence Interval for Mean | Lower Bound | | 6,3381 | |
| | | Upper Bound | | 8,6119 | |
| | 5% Trimmed Mean | | | 7,2361 | |
| | Median | | | 7,0000 | |
| | Variance | | | 26,101 | |
| | Std. Deviation | | | 5,10888 | |
| | Minimum | | | ,00 | |
| | Maximum | | | 22,00 | |
| | Range | | | 22,00 | |
| | Interquartile Range | | | 7,00 | |
| | Skewness | | | ,625 | ,269 |
| | Kurtosis | | | -,112 | ,532 |

```
 SPLIT FILE OFF.
T-TEST GROUPS=Grau_Endometriodo_Cat(1 2)
  /MISSING=ANALYSIS
  /VARIABLES=idade tempo_ACO
  /CRITERIA=CI(.95).
```

# T-Test

**Notes**

| | | |
|---|---|---|
| Output Created | | 08-MAY-2020 09:34:08 |
| Comments | | |
| Input | Data | \\Mac\iCloud\Lavoro\FMABC\Orientações\Orientações\finite\Marina Rodrigues - Caio e Fabia\Marina Farias\data_article_5.sav |
| | Active Dataset | InsiemeDati1 |
| | Filter | <none> |
| | Weight | <none> |
| | Split File | <none> |
| | N of Rows in Working Data File | 106 |
| Missing Value Handling | Definition of Missing | User defined missing values are treated as missing. |
| | Cases Used | Statistics for each analysis are based on the cases with no missing or out-of-range data for any variable in the analysis. |
| Syntax | | T-TEST GROUPS=Grau_Endometriodo_Cat (1 2)<br>/MISSING=ANALYSIS<br>/VARIABLES=idade tempo_ACO<br>/CRITERIA=CI(.95). |
| Resources | Processor Time | 00:00:00,00 |
| | Elapsed Time | 00:00:00,01 |

```
[InsiemeDati1] \\Mac\iCloud\Lavoro\FMABC\Orientações\Orientações\finite\Marin
a Rodrigues - Caio e Fabia\Marina Farias\data_article_5.sav
```

**Group Statistics**

| | Grau_Endometriodo_Cat | N | Mean | Std. Deviation | Std. Error Mean |
|---|---|---|---|---|---|
| idade | Grau I/II | 26 | 35,27 | 3,639 | ,714 |
| | Grau III/IV | 80 | 34,04 | 3,274 | ,366 |
| tempo_ACO | Grau I/II | 26 | 4,8038 | 4,31030 | ,84532 |
| | Grau III/IV | 80 | 7,4750 | 5,10888 | ,57119 |

**Independent Samples Test**

| | | Levene's Test for Equality of Variances | | t-test for Equality of Means | |
|---|---|---|---|---|---|
| | | F | Sig. | t | df |
| idade | Equal variances assumed | ,126 | ,723 | 1,621 | 104 |
| | Equal variances not assumed | | | 1,536 | 39,028 |
| tempo_ACO | Equal variances assumed | 1,639 | ,203 | -2,401 | 104 |
| | Equal variances not assumed | | | -2,618 | 49,758 |

**Independent Samples Test**

| | | t-test for Equality of Means | | |
|---|---|---|---|---|
| | | Sig. (2-tailed) | Mean Difference | Std. Error Difference |
| idade | Equal variances assumed | ,108 | 1,232 | ,760 |
| | Equal variances not assumed | ,133 | 1,232 | ,802 |
| tempo_ACO | Equal variances assumed | ,018 | -2,67115 | 1,11265 |
| | Equal variances not assumed | ,012 | -2,67115 | 1,02021 |

**Independent Samples Test**

| | | t-test for Equality of Means | |
|---|---|---|---|
| | | 95% Confidence Interval of the Difference | |
| | | Lower | Upper |
| idade | Equal variances assumed | -,275 | 2,738 |
| | Equal variances not assumed | -,391 | 2,854 |
| tempo_ACO | Equal variances assumed | -4,87757 | -,46473 |
| | Equal variances not assumed | -4,72055 | -,62176 |

```
 *Nonparametric Tests: Independent Samples.
NPTESTS
  /INDEPENDENT TEST (temp_infert menarca) GROUP (Grau_Endometriodo_Cat) MANN_WHITNEY
  /MISSING SCOPE=ANALYSIS USERMISSING=EXCLUDE
  /CRITERIA ALPHA=0.05  CILEVEL=95.
```

# Nonparametric Tests

**Notes**

| | | |
|---|---|---|
| Output Created | | 08-MAY-2020 09:35:39 |
| Comments | | |
| Input | Data | \\Mac\iCloud\Lavoro\FMABC\Orientações\Orientações\finite\Marina Rodrigues - Caio e Fabia\Marina Farias\data_article_5.sav |
| | Active Dataset | InsiemeDati1 |
| | Filter | <none> |
| | Weight | <none> |
| | Split File | <none> |
| | N of Rows in Working Data File | 106 |
| Syntax | | NPTESTS /INDEPENDENT TEST (temp_infert menarca) GROUP (Grau_Endometriodo_Cat) MANN_WHITNEY /MISSING SCOPE=ANALYSIS USERMISSING=EXCLUDE /CRITERIA ALPHA=0.05 CILEVEL=95. |
| Resources | Processor Time | 00:00:00,11 |
| | Elapsed Time | 00:00:00,23 |

```
[InsiemeDati1] \\Mac\iCloud\Lavoro\FMABC\Orientações\Orientações\finite\Marin
a Rodrigues - Caio e Fabia\Marina Farias\data_article_5.sav
```

## Hypothesis Test Summary

| | Null Hypothesis | Test | Sig. | Decision |
|---|---|---|---|---|
| 1 | The distribution of Tempo de infertilidade em anos is the same across categories of Grau Endometriose cat. | Independent-Samples Mann-Whitney U Test | ,654 | Retain the null hypothesis. |
| 2 | The distribution of idade menarca is the same across categories of Grau Endometriose cat. | Independent-Samples Mann-Whitney U Test | ,254 | Retain the null hypothesis. |

Asymptotic significances are displayed. The significance level is ,05.

```
 SORT CASES  BY Grau_Endometriodo_Cat.
SPLIT FILE LAYERED BY Grau_Endometriodo_Cat.
FREQUENCIES VARIABLES=tipo_infert anticoncecpcional_prev dismenorreia grau_dor dispaurenia dor_
  /ORDER=ANALYSIS.
```

# Frequencies

**Notes**

| | | |
|---|---|---|
| Output Created | | 08-MAY-2020 09:37:28 |
| Comments | | |
| Input | Data | \\Mac\iCloud\Lavoro\FMABC\Orientações\Orientações\finite\Marina Rodrigues - Caio e Fabia\Marina Farias\data_article_5.sav |
| | Active Dataset | InsiemeDati1 |
| | Filter | <none> |
| | Weight | <none> |
| | Split File | Grau_Endometriodo_Cat |
| | N of Rows in Working Data File | 106 |
| Missing Value Handling | Definition of Missing | User-defined missing values are treated as missing. |
| | Cases Used | Statistics are based on all cases with valid data. |
| Syntax | | FREQUENCIES VARIABLES=tipo_infert anticoncecpcional_prev dismenorreia grau_dor dispaurenia dor_fora_menst alt_intestinais alt_urinaria atend_endom atend_infert historico_aborto /ORDER=ANALYSIS. |
| Resources | Processor Time | 00:00:00,02 |
| | Elapsed Time | 00:00:00,01 |

[InsiemeDati1] \\Mac\iCloud\Lavoro\FMABC\Orientações\Orientações\finite\Marina Rodrigues - Caio e Fabia\Marina Farias\data_article_5.sav

**Statistics**

| Grau_Endometriodo_Cat | | | tipo_infert | anticoncecpcional_prev | dismenorreia | grau_dor | dispaurenia |
|---|---|---|---|---|---|---|---|
| Grau I/II | N | Valid | 26 | 26 | 26 | 26 | 26 |
| | | Missing | 0 | 0 | 0 | 0 | 0 |
| Grau III/IV | N | Valid | 80 | 80 | 80 | 80 | 80 |
| | | Missing | 0 | 0 | 0 | 0 | 0 |

**Statistics**

| Grau_Endometriodo_Cat | | | dor_fora_menst | alt_intestinais | alt_urinaria | atend_endom |
|---|---|---|---|---|---|---|
| Grau I/II | N | Valid | 26 | 26 | 26 | 26 |
| | | Missing | 0 | 0 | 0 | 0 |
| Grau III/IV | N | Valid | 80 | 80 | 80 | 80 |
| | | Missing | 0 | 0 | 0 | 0 |

**Statistics**

| Grau_Endometriodo_Cat | | | atend_infert | historico_aborto |
|---|---|---|---|---|
| Grau I/II | N | Valid | 26 | 26 |
| | | Missing | 0 | 0 |
| Grau III/IV | N | Valid | 80 | 80 |
| | | Missing | 0 | 0 |

# Frequency Table

**tipo_infert**

| Grau_Endometriodo_Cat | | | Frequency | Percent | Valid Percent | Cumulative Percent |
|---|---|---|---|---|---|---|
| Grau I/II | Valid | Primária | 23 | 88,5 | 88,5 | 88,5 |
| | | Secundária | 3 | 11,5 | 11,5 | 100,0 |
| | | Total | 26 | 100,0 | 100,0 | |
| Grau III/IV | Valid | Primária | 69 | 86,3 | 86,3 | 86,3 |
| | | Secundária | 11 | 13,8 | 13,8 | 100,0 |
| | | Total | 80 | 100,0 | 100,0 | |

**anticoncecpcional_prev**

| Grau_Endometriodo_Cat | | | Frequency | Percent | Valid Percent | Cumulative Percent |
|---|---|---|---|---|---|---|
| Grau I/II | Valid | nao | 2 | 7,7 | 7,7 | 7,7 |
| | | sim | 24 | 92,3 | 92,3 | 100,0 |
| | | Total | 26 | 100,0 | 100,0 | |
| Grau III/IV | Valid | nao | 4 | 5,0 | 5,0 | 5,0 |
| | | sim | 76 | 95,0 | 95,0 | 100,0 |
| | | Total | 80 | 100,0 | 100,0 | |

**dismenorreia**

| Grau_Endometriodo_Cat | | | Frequency | Percent | Valid Percent | Cumulative Percent |
|---|---|---|---|---|---|---|
| Grau I/II | Valid | Ausente | 4 | 15,4 | 15,4 | 15,4 |
| | | Primária | 10 | 38,5 | 38,5 | 53,8 |
| | | Secundária | 12 | 46,2 | 46,2 | 100,0 |
| | | Total | 26 | 100,0 | 100,0 | |
| Grau III/IV | Valid | Ausente | 16 | 20,0 | 20,0 | 20,0 |
| | | Primária | 27 | 33,8 | 33,8 | 53,8 |
| | | Secundária | 37 | 46,3 | 46,3 | 100,0 |
| | | Total | 80 | 100,0 | 100,0 | |

**grau_dor**

| Grau_Endometriodo_Cat | | | Frequency | Percent | Valid Percent | Cumulative Percent |
|---|---|---|---|---|---|---|
| Grau I/II | Valid | Ausente | 5 | 19,2 | 19,2 | 19,2 |
| | | Leve | 2 | 7,7 | 7,7 | 26,9 |
| | | Moderada | 9 | 34,6 | 34,6 | 61,5 |
| | | Severa | 8 | 30,8 | 30,8 | 92,3 |
| | | Incapacitante | 2 | 7,7 | 7,7 | 100,0 |
| | | Total | 26 | 100,0 | 100,0 | |
| Grau III/IV | Valid | Ausente | 12 | 15,0 | 15,0 | 15,0 |
| | | Leve | 6 | 7,5 | 7,5 | 22,5 |
| | | Moderada | 21 | 26,3 | 26,3 | 48,8 |
| | | Severa | 33 | 41,3 | 41,3 | 90,0 |
| | | Incapacitante | 8 | 10,0 | 10,0 | 100,0 |
| | | Total | 80 | 100,0 | 100,0 | |

**dispaurenia**

| Grau_Endometriodo_Cat | | | Frequency | Percent | Valid Percent |
|---|---|---|---|---|---|
| Grau I/II | Valid | Ausente | 14 | 53,8 | 53,8 |
| | | Penetração | 4 | 15,4 | 15,4 |
| | | Profundidade | 7 | 26,9 | 26,9 |
| | | Penetraçao e Profundiddade | 1 | 3,8 | 3,8 |
| | | Total | 26 | 100,0 | 100,0 |
| Grau III/IV | Valid | Ausente | 42 | 52,5 | 52,5 |
| | | Penetração | 9 | 11,3 | 11,3 |
| | | Profundidade | 17 | 21,3 | 21,3 |
| | | Penetraçao e Profundiddade | 12 | 15,0 | 15,0 |
| | | Total | 80 | 100,0 | 100,0 |

**dispaurenia**

| Grau_Endometriodo_Cat | | | Cumulative Percent |
|---|---|---|---|
| Grau I/II | Valid | Ausente | 53,8 |
| | | Penetração | 69,2 |
| | | Profundidade | 96,2 |
| | | Penetraçao e Profundiddade | 100,0 |
| | | Total | |
| Grau III/IV | Valid | Ausente | 52,5 |
| | | Penetração | 63,8 |
| | | Profundidade | 85,0 |
| | | Penetraçao e Profundiddade | 100,0 |
| | | Total | |

**dor_fora_menst**

| Grau_Endometriodo_Cat | | | Frequency | Percent | Valid Percent | Cumulative Percent |
|---|---|---|---|---|---|---|
| Grau I/II | Valid | não | 19 | 73,1 | 73,1 | 73,1 |
| | | sim | 7 | 26,9 | 26,9 | 100,0 |
| | | Total | 26 | 100,0 | 100,0 | |
| Grau III/IV | Valid | não | 66 | 82,5 | 82,5 | 82,5 |
| | | sim | 14 | 17,5 | 17,5 | 100,0 |
| | | Total | 80 | 100,0 | 100,0 | |

**alt_intestinais**

| Grau_Endometriodo_Cat | | | Frequency | Percent | Valid Percent | Cumulative Percent |
|---|---|---|---|---|---|---|
| Grau I/II | Valid | não | 6 | 23,1 | 23,1 | 23,1 |
| | | sim | 20 | 76,9 | 76,9 | 100,0 |
| | | Total | 26 | 100,0 | 100,0 | |
| Grau III/IV | Valid | não | 23 | 28,8 | 28,8 | 28,8 |
| | | sim | 57 | 71,3 | 71,3 | 100,0 |
| | | Total | 80 | 100,0 | 100,0 | |

**alt_urinaria**

| Grau_Endometriodo_Cat | | | Frequency | Percent | Valid Percent | Cumulative Percent |
|---|---|---|---|---|---|---|
| Grau I/II | Valid | não | 25 | 96,2 | 96,2 | 96,2 |
| | | sim | 1 | 3,8 | 3,8 | 100,0 |
| | | Total | 26 | 100,0 | 100,0 | |
| Grau III/IV | Valid | não | 76 | 95,0 | 95,0 | 95,0 |
| | | sim | 4 | 5,0 | 5,0 | 100,0 |
| | | Total | 80 | 100,0 | 100,0 | |

**atend_endom**

| Grau_Endometriodo_Cat | | | Frequency | Percent | Valid Percent | Cumulative Percent |
|---|---|---|---|---|---|---|
| Grau I/II | Valid | 1 a 4 vezes | 17 | 65,4 | 65,4 | 65,4 |
| | | 5 a 9 vezes | 7 | 26,9 | 26,9 | 92,3 |
| | | 10 a 15 vezes | 1 | 3,8 | 3,8 | 96,2 |
| | | 16 a 20 vezes | 1 | 3,8 | 3,8 | 100,0 |
| | | Total | 26 | 100,0 | 100,0 | |
| Grau III/IV | Valid | nenhuma vez | 2 | 2,5 | 2,5 | 2,5 |
| | | 1 a 4 vezes | 43 | 53,8 | 53,8 | 56,3 |
| | | 5 a 9 vezes | 16 | 20,0 | 20,0 | 76,3 |
| | | 10 a 15 vezes | 12 | 15,0 | 15,0 | 91,3 |
| | | 16 a 20 vezes | 1 | 1,3 | 1,3 | 92,5 |
| | | acima de 20 vezes | 6 | 7,5 | 7,5 | 100,0 |
| | | Total | 80 | 100,0 | 100,0 | |

**atend_infert**

| Grau_Endometriodo_Cat | | | Frequency | Percent | Valid Percent | Cumulative Percent |
|---|---|---|---|---|---|---|
| Grau I/II | Valid | 1 a 4 vezes | 23 | 88,5 | 88,5 | 88,5 |
| | | 10 a 15 vezes | 2 | 7,7 | 7,7 | 96,2 |
| | | acima de 20 vezes | 1 | 3,8 | 3,8 | 100,0 |
| | | Total | 26 | 100,0 | 100,0 | |
| Grau III/IV | Valid | 1 a 4 vezes | 62 | 77,5 | 77,5 | 77,5 |
| | | 5 a 9 vezes | 11 | 13,8 | 13,8 | 91,3 |
| | | 10 a 15 vezes | 7 | 8,8 | 8,8 | 100,0 |
| | | Total | 80 | 100,0 | 100,0 | |

**historico_aborto**

| Grau_Endometriodo_Cat | | | Frequency | Percent | Valid Percent | Cumulative Percent |
|---|---|---|---|---|---|---|
| Grau I/II | Valid | nao | 20 | 76,9 | 76,9 | 76,9 |
| | | sim | 6 | 23,1 | 23,1 | 100,0 |
| | | Total | 26 | 100,0 | 100,0 | |
| Grau III/IV | Valid | nao | 66 | 82,5 | 82,5 | 82,5 |
| | | sim | 14 | 17,5 | 17,5 | 100,0 |
| | | Total | 80 | 100,0 | 100,0 | |

```
 SPLIT FILE OFF.
CROSSTABS
  /TABLES=Grau_Endometriodo_Cat BY tipo_infert historico_aborto anticoncecpcional_prev grau_dor
  /FORMAT=AVALUE TABLES
```

```
/STATISTICS=CHISQ
/CELLS=COUNT
/COUNT ROUND CELL.
```

# Crosstabs

**Notes**

| | | |
|---|---|---|
| Output Created | | 08-MAY-2020 09:59:15 |
| Comments | | |
| Input | Data | \\Mac\iCloud\Lavoro\FMABC\Orientações\Orientações\finite\Marina Rodrigues - Caio e Fabia\Marina Farias\data_article_5.sav |
| | Active Dataset | InsiemeDati1 |
| | Filter | <none> |
| | Weight | <none> |
| | Split File | <none> |
| | N of Rows in Working Data File | 106 |
| Missing Value Handling | Definition of Missing | User-defined missing values are treated as missing. |
| | Cases Used | Statistics for each table are based on all the cases with valid data in the specified range(s) for all variables in each table. |
| Syntax | | CROSSTABS /TABLES=Grau_Endometriodo_Cat BY tipo_infert historico_aborto anticoncecpcional_prev grau_dor dismenorreia dispaurenia dor_fora_menst alt_intestinais alt_urinaria /FORMAT=AVALUE TABLES /STATISTICS=CHISQ /CELLS=COUNT /COUNT ROUND CELL. |
| Resources | Processor Time | 00:00:00,02 |
| | Elapsed Time | 00:00:00,02 |
| | Dimensions Requested | 2 |
| | Cells Available | 174734 |

```
[InsiemeDati1] \\Mac\iCloud\Lavoro\FMABC\Orientações\Orientações\finite\Marina Rodrigues - Caio e Fabia\Marina Farias\data_article_5.sav
```

**Case Processing Summary**

| | Cases | | | | |
|---|---|---|---|---|---|
| | Valid | | Missing | | Total |
| | N | Percent | N | Percent | N |
| Grau_Endometriodo_Cat * tipo_infert | 106 | 100,0% | 0 | 0,0% | 106 |
| Grau_Endometriodo_Cat * historico_aborto | 106 | 100,0% | 0 | 0,0% | 106 |
| Grau_Endometriodo_Cat * anticoncecpcional_prev | 106 | 100,0% | 0 | 0,0% | 106 |
| Grau_Endometriodo_Cat * grau_dor | 106 | 100,0% | 0 | 0,0% | 106 |
| Grau_Endometriodo_Cat * dismenorreia | 106 | 100,0% | 0 | 0,0% | 106 |
| Grau_Endometriodo_Cat * dispaurenia | 106 | 100,0% | 0 | 0,0% | 106 |
| Grau_Endometriodo_Cat * dor_fora_menst | 106 | 100,0% | 0 | 0,0% | 106 |
| Grau_Endometriodo_Cat * alt_intestinais | 106 | 100,0% | 0 | 0,0% | 106 |
| Grau_Endometriodo_Cat * alt_urinaria | 106 | 100,0% | 0 | 0,0% | 106 |

**Case Processing Summary**

| | Cases |
|---|---|
| | Total |
| | Percent |
| Grau_Endometriodo_Cat * tipo_infert | 100,0% |
| Grau_Endometriodo_Cat * historico_aborto | 100,0% |
| Grau_Endometriodo_Cat * anticoncecpcional_prev | 100,0% |
| Grau_Endometriodo_Cat * grau_dor | 100,0% |
| Grau_Endometriodo_Cat * dismenorreia | 100,0% |
| Grau_Endometriodo_Cat * dispaurenia | 100,0% |
| Grau_Endometriodo_Cat * dor_fora_menst | 100,0% |
| Grau_Endometriodo_Cat * alt_intestinais | 100,0% |
| Grau_Endometriodo_Cat * alt_urinaria | 100,0% |

# Grau_Endometriodo_Cat * tipo_infert

**Crosstab**

Count

| | | tipo_infert | | Total |
|---|---|---|---|---|
| | | Primária | Secundária | |
| Grau_Endometriodo_Cat | Grau I/II | 23 | 3 | 26 |
| | Grau III/IV | 69 | 11 | 80 |
| Total | | 92 | 14 | 106 |

**Chi-Square Tests**

| | Value | df | Asymp. Sig. (2-sided) | Exact Sig. (2-sided) | Exact Sig. (1-sided) |
|---|---|---|---|---|---|
| Pearson Chi-Square | ,084[a] | 1 | ,772 | | |
| Continuity Correction[b] | ,000 | 1 | 1,000 | | |
| Likelihood Ratio | ,086 | 1 | ,769 | | |
| Fisher's Exact Test | | | | 1,000 | ,535 |
| Linear-by-Linear Association | ,083 | 1 | ,773 | | |
| N of Valid Cases | 106 | | | | |

a. 1 cells (25,0%) have expected count less than 5. The minimum expected count is 3,43.

b. Computed only for a 2x2 table

## Grau_Endometriodo_Cat * historico_aborto

**Crosstab**

Count

| | | historico_aborto | | Total |
|---|---|---|---|---|
| | | nao | sim | |
| Grau_Endometriodo_Cat | Grau I/II | 20 | 6 | 26 |
| | Grau III/IV | 66 | 14 | 80 |
| Total | | 86 | 20 | 106 |

**Chi-Square Tests**

| | Value | df | Asymp. Sig. (2-sided) | Exact Sig. (2-sided) | Exact Sig. (1-sided) |
|---|---|---|---|---|---|
| Pearson Chi-Square | ,399[a] | 1 | ,528 | | |
| Continuity Correction[b] | ,118 | 1 | ,732 | | |
| Likelihood Ratio | ,385 | 1 | ,535 | | |
| Fisher's Exact Test | | | | ,568 | ,356 |
| Linear-by-Linear Association | ,395 | 1 | ,530 | | |
| N of Valid Cases | 106 | | | | |

a. 1 cells (25,0%) have expected count less than 5. The minimum expected count is 4,91.

b. Computed only for a 2x2 table

## Grau_Endometriodo_Cat * anticoncecpcional_prev

**Crosstab**

Count

| | | anticoncecpcional_prev | | Total |
|---|---|---|---|---|
| | | nao | sim | |
| Grau_Endometriodo_Cat | Grau I/II | 2 | 24 | 26 |
| | Grau III/IV | 4 | 76 | 80 |
| Total | | 6 | 100 | 106 |

**Chi-Square Tests**

| | Value | df | Asymp. Sig. (2-sided) | Exact Sig. (2-sided) | Exact Sig. (1-sided) |
|---|---|---|---|---|---|
| Pearson Chi-Square | ,266[a] | 1 | ,606 | | |
| Continuity Correction[b] | ,001 | 1 | ,978 | | |
| Likelihood Ratio | ,250 | 1 | ,617 | | |
| Fisher's Exact Test | | | | ,634 | ,457 |
| Linear-by-Linear Association | ,264 | 1 | ,607 | | |
| N of Valid Cases | 106 | | | | |

a. 2 cells (50,0%) have expected count less than 5. The minimum expected count is 1,47.

b. Computed only for a 2x2 table

## Grau_Endometriodo_Cat * grau_dor

**Crosstab**

Count

| | | grau_dor | | | | |
|---|---|---|---|---|---|---|
| | | Ausente | Leve | Moderada | Severa | Incapacitante |
| Grau_Endometriodo_Cat | Grau I/II | 5 | 2 | 9 | 8 | 2 |
| | Grau III/IV | 12 | 6 | 21 | 33 | 8 |
| Total | | 17 | 8 | 30 | 41 | 10 |

**Crosstab**

Count

| | | Total |
|---|---|---|
| Grau_Endometriodo_Cat | Grau I/II | 26 |
| | Grau III/IV | 80 |
| Total | | 106 |

**Chi-Square Tests**

| | Value | df | Asymp. Sig. (2-sided) |
|---|---|---|---|
| Pearson Chi-Square | 1,373[a] | 4 | ,849 |
| Likelihood Ratio | 1,377 | 4 | ,848 |
| Linear-by-Linear Association | ,757 | 1 | ,384 |
| N of Valid Cases | 106 | | |

a. 3 cells (30,0%) have expected count less than 5. The minimum expected count is 1,96.

## Grau_Endometriodo_Cat * dismenorreia

**Crosstab**

Count

| | | dismenorreia | | | Total |
|---|---|---|---|---|---|
| | | Ausente | Primária | Secundária | |
| Grau_Endometriodo_Cat | Grau I/II | 4 | 10 | 12 | 26 |
| | Grau III/IV | 16 | 27 | 37 | 80 |
| Total | | 20 | 37 | 49 | 106 |

**Chi-Square Tests**

| | Value | df | Asymp. Sig. (2-sided) |
|---|---|---|---|
| Pearson Chi-Square | ,346[a] | 2 | ,841 |
| Likelihood Ratio | ,354 | 2 | ,838 |
| Linear-by-Linear Association | ,069 | 1 | ,793 |
| N of Valid Cases | 106 | | |

a. 1 cells (16,7%) have expected count less than 5. The minimum expected count is 4,91.

## Grau_Endometriodo_Cat * dispaurenia

**Crosstab**

Count

| | | dispaurenia | | | |
|---|---|---|---|---|---|
| | | Ausente | Penetração | Profundidade | Penetraçao e Profundiddad e |
| Grau_Endometriodo_Cat | Grau I/II | 14 | 4 | 7 | 1 |
| | Grau III/IV | 42 | 9 | 17 | 12 |
| Total | | 56 | 13 | 24 | 13 |

**Crosstab**

Count

| | | Total |
|---|---|---|
| Grau_Endometriodo_Cat | Grau I/II | 26 |
| | Grau III/IV | 80 |
| Total | | 106 |

**Chi-Square Tests**

| | Value | df | Asymp. Sig. (2-sided) |
|---|---|---|---|
| Pearson Chi-Square | 2,550[a] | 3 | ,466 |
| Likelihood Ratio | 3,048 | 3 | ,384 |
| Linear-by-Linear Association | ,506 | 1 | ,477 |
| N of Valid Cases | 106 | | |

a. 2 cells (25,0%) have expected count less than 5. The minimum expected count is 3,19.

# Grau_Endometriodo_Cat * dor_fora_menst

**Crosstab**

Count

| | | dor_fora_menst | | Total |
|---|---|---|---|---|
| | | não | sim | |
| Grau_Endometriodo_Cat | Grau I/II | 19 | 7 | 26 |
| | Grau III/IV | 66 | 14 | 80 |
| Total | | 85 | 21 | 106 |

**Chi-Square Tests**

| | Value | df | Asymp. Sig. (2-sided) | Exact Sig. (2-sided) | Exact Sig. (1-sided) |
|---|---|---|---|---|---|
| Pearson Chi-Square | 1,097[a] | 1 | ,295 | | |
| Continuity Correction[b] | ,584 | 1 | ,445 | | |
| Likelihood Ratio | 1,043 | 1 | ,307 | | |
| Fisher's Exact Test | | | | ,395 | ,219 |
| Linear-by-Linear Association | 1,086 | 1 | ,297 | | |
| N of Valid Cases | 106 | | | | |

a. 0 cells (,0%) have expected count less than 5. The minimum expected count is 5,15.

b. Computed only for a 2x2 table

# Grau_Endometriodo_Cat * alt_intestinais

**Crosstab**

Count

| | | alt_intestinais | | Total |
|---|---|---|---|---|
| | | não | sim | |
| Grau_Endometriodo_Cat | Grau I/II | 6 | 20 | 26 |
| | Grau III/IV | 23 | 57 | 80 |
| Total | | 29 | 77 | 106 |

**Chi-Square Tests**

| | Value | df | Asymp. Sig. (2-sided) | Exact Sig. (2-sided) | Exact Sig. (1-sided) |
|---|---|---|---|---|---|
| Pearson Chi-Square | ,318[a] | 1 | ,573 | | |
| Continuity Correction[b] | ,096 | 1 | ,756 | | |
| Likelihood Ratio | ,326 | 1 | ,568 | | |
| Fisher's Exact Test | | | | ,624 | ,385 |
| Linear-by-Linear Association | ,315 | 1 | ,575 | | |
| N of Valid Cases | 106 | | | | |

a. 0 cells (,0%) have expected count less than 5. The minimum expected count is 7,11.

b. Computed only for a 2x2 table

# Grau_Endometriodo_Cat * alt_urinaria

**Crosstab**

Count

| | | alt_urinaria | | Total |
|---|---|---|---|---|
| | | não | sim | |
| Grau_Endometriodo_Cat | Grau I/II | 25 | 1 | 26 |
| | Grau III/IV | 76 | 4 | 80 |
| Total | | 101 | 5 | 106 |

**Chi-Square Tests**

| | Value | df | Asymp. Sig. (2-sided) | Exact Sig. (2-sided) | Exact Sig. (1-sided) |
|---|---|---|---|---|---|
| Pearson Chi-Square | ,058[a] | 1 | ,809 | | |
| Continuity Correction[b] | ,000 | 1 | 1,000 | | |
| Likelihood Ratio | ,061 | 1 | ,805 | | |
| Fisher's Exact Test | | | | 1,000 | ,643 |
| Linear-by-Linear Association | ,058 | 1 | ,810 | | |
| N of Valid Cases | 106 | | | | |

a. 2 cells (50,0%) have expected count less than 5. The minimum expected count is 1,23.

b. Computed only for a 2x2 table

```
SAVE OUTFILE='\\Mac\iCloud\Lavoro\FMABC\Orientações\Orientações\finite\Marina Rodrigues - Caio
    'Fabia\Marina Farias\data_article_6.sav'
  /COMPRESSED.
DATASET ACTIVATE InsiemeDati1.

SAVE OUTFILE='\\Mac\iCloud\Lavoro\FMABC\Orientações\Orientações\finite\Marina Rodrigues - Caio
    'Fabia\Marina Farias\data_article_6.sav'
  /COMPRESSED.
T-TEST GROUPS=Grau_Endometriodo_Cat(1 2)
  /MISSING=ANALYSIS
  /VARIABLES=estado_geral vitalidade asp_sociais saude_mental
  /CRITERIA=CI(.95).
```

# T-Test

**Notes**

| | | |
|---|---|---|
| Output Created | | 08-MAY-2020 10:21:20 |
| Comments | | |
| Input | Data | \\Mac\iCloud\Lavoro\FMABC\Orientações\Orientações\finite\Marina Rodrigues - Caio e Fabia\Marina Farias\data_article_6.sav |
| | Active Dataset | InsiemeDati1 |
| | Filter | <none> |
| | Weight | <none> |
| | Split File | <none> |
| | N of Rows in Working Data File | 106 |
| Missing Value Handling | Definition of Missing | User defined missing values are treated as missing. |
| | Cases Used | Statistics for each analysis are based on the cases with no missing or out-of-range data for any variable in the analysis. |
| Syntax | | T-TEST GROUPS=Grau_Endometriodo_Cat(1 2) /MISSING=ANALYSIS /VARIABLES=estado_geral vitalidade asp_sociais saude_mental /CRITERIA=CI(.95). |
| Resources | Processor Time | 00:00:00,02 |
| | Elapsed Time | 00:00:00,01 |

[InsiemeDati1] \\Mac\iCloud\Lavoro\FMABC\Orientações\Orientações\finite\Marina Rodrigues - Caio e Fabia\Marina Farias\data_article_6.sav

**Group Statistics**

| | Grau_Endometriodo_Cat | N | Mean | Std. Deviation | Std. Error Mean |
|---|---|---|---|---|---|
| estado_geral | Grau I/II | 26 | 58,692308 | 16,5620512 | 3,2480855 |
| | Grau III/IV | 80 | 60,537500 | 17,5743943 | 1,9648770 |
| vitalidade | Grau I/II | 26 | 54,42 | 14,719 | 2,887 |
| | Grau III/IV | 80 | 56,24 | 11,380 | 1,272 |
| asp_sociais | Grau I/II | 26 | 66,346 | 26,4029 | 5,1780 |
| | Grau III/IV | 80 | 66,200 | 23,5781 | 2,6361 |
| saude_mental | Grau I/II | 26 | 59,54 | 21,176 | 4,153 |
| | Grau III/IV | 80 | 59,23 | 18,525 | 2,071 |

**Independent Samples Test**

| | | Levene's Test for Equality of Variances | | t-test for Equality of Means | |
|---|---|---|---|---|---|
| | | F | Sig. | t | df |
| estado_geral | Equal variances assumed | ,006 | ,940 | -,471 | 104 |
| | Equal variances not assumed | | | -,486 | 44,749 |
| vitalidade | Equal variances assumed | 3,218 | ,076 | -,655 | 104 |
| | Equal variances not assumed | | | -,575 | 35,236 |
| asp_sociais | Equal variances assumed | ,676 | ,413 | ,027 | 104 |
| | Equal variances not assumed | | | ,025 | 38,813 |
| saude_mental | Equal variances assumed | 1,924 | ,168 | ,072 | 104 |
| | Equal variances not assumed | | | ,068 | 38,234 |

**Independent Samples Test**

| | | t-test for Equality of Means | | |
|---|---|---|---|---|
| | | Sig. (2-tailed) | Mean Difference | Std. Error Difference |
| estado_geral | Equal variances assumed | ,638 | -1,8451923 | 3,9136429 |
| | Equal variances not assumed | ,629 | -1,8451923 | 3,7961561 |
| vitalidade | Equal variances assumed | ,514 | -1,814 | 2,769 |
| | Equal variances not assumed | ,569 | -1,814 | 3,155 |
| asp_sociais | Equal variances assumed | ,979 | ,1462 | 5,4827 |
| | Equal variances not assumed | ,980 | ,1462 | 5,8104 |
| saude_mental | Equal variances assumed | ,942 | ,313 | 4,333 |
| | Equal variances not assumed | ,946 | ,313 | 4,641 |

**Independent Samples Test**

| | | t-test for Equality of Means | |
|---|---|---|---|
| | | 95% Confidence Interval of the Difference | |
| | | Lower | Upper |
| estado_geral | Equal variances assumed | -9,6060931 | 5,9157085 |
| | Equal variances not assumed | -9,4922281 | 5,8018435 |
| vitalidade | Equal variances assumed | -7,305 | 3,677 |
| | Equal variances not assumed | -8,217 | 4,588 |
| asp_sociais | Equal variances assumed | -10,7263 | 11,0187 |
| | Equal variances not assumed | -11,6084 | 11,9007 |
| saude_mental | Equal variances assumed | -8,280 | 8,907 |
| | Equal variances not assumed | -9,079 | 9,706 |

```
 *Nonparametric Tests: Independent Samples.
NPTESTS
  /INDEPENDENT TEST (cap_func lim_fisico dor lim_emocional) GROUP (Grau_Endometriodo_Cat) MANN_
  /MISSING SCOPE=ANALYSIS USERMISSING=EXCLUDE
  /CRITERIA ALPHA=0.05  CILEVEL=95.
```

## Nonparametric Tests

**Notes**

| | | |
|---|---|---|
| Output Created | | 08-MAY-2020 10:21:54 |
| Comments | | |
| Input | Data | \\Mac\iCloud\Lavoro\FMABC\Orientações\Orientações\finite\Marina Rodrigues - Caio e Fabia\Marina Farias\data_article_6.sav |
| | Active Dataset | InsiemeDati1 |
| | Filter | <none> |
| | Weight | <none> |
| | Split File | <none> |
| | N of Rows in Working Data File | 106 |
| Syntax | | NPTESTS /INDEPENDENT TEST (cap_func lim_fisico dor lim_emocional) GROUP (Grau_Endometriodo_Cat) MANN_WHITNEY /MISSING SCOPE=ANALYSIS USERMISSING=EXCLUDE /CRITERIA ALPHA=0.05 CILEVEL=95. |
| Resources | Processor Time | 00:00:00,09 |
| | Elapsed Time | 00:00:00,08 |

```
[InsiemeDati1] \\Mac\iCloud\Lavoro\FMABC\Orientações\Orientações\finite\Marin
a Rodrigues - Caio e Fabia\Marina Farias\data_article_6.sav
```

## Hypothesis Test Summary

| | Null Hypothesis | Test | Sig. | Decision |
|---|---|---|---|---|
| 1 | The distribution of SF-36 - capacidade funcional is the same across categories of Grau Endometriose cat. | Independent-Samples Mann-Whitney U Test | ,708 | Retain the null hypothesis. |
| 2 | The distribution of SF-36 - limitacao fisica is the same across categories of Grau Endometriose cat. | Independent-Samples Mann-Whitney U Test | ,794 | Retain the null hypothesis. |
| 3 | The distribution of SF-36 - dor is the same across categories of Grau Endometriose cat. | Independent-Samples Mann-Whitney U Test | ,352 | Retain the null hypothesis. |
| 4 | The distribution of SF-36 - limites emocionais is the same across categories of Grau Endometriose cat. | Independent-Samples Mann-Whitney U Test | ,360 | Retain the null hypothesis. |

Asymptotic significances are displayed. The significance level is ,05.

```
 SORT CASES  BY Grau_Endometriodo_Cat.
SPLIT FILE LAYERED BY Grau_Endometriodo_Cat.
EXAMINE VARIABLES=cap_func lim_fisico dor lim_emocional estado_geral vitalidade asp_sociais sau
  /PLOT NONE
  /STATISTICS DESCRIPTIVES
  /CINTERVAL 95
  /MISSING LISTWISE
  /NOTOTAL.
```

# Explore

**Notes**

| | | |
|---|---|---|
| Output Created | | 08-MAY-2020 10:40:36 |
| Comments | | |
| Input | Data | \\Mac\iCloud\Lavoro\FMABC\Orientações\Orientações\finite\Marina Rodrigues - Caio e Fabia\Marina Farias\data_article_6.sav |
| | Active Dataset | InsiemeDati1 |
| | Filter | <none> |
| | Weight | <none> |
| | Split File | Grau_Endometriodo_Cat |
| | N of Rows in Working Data File | 106 |
| Missing Value Handling | Definition of Missing | User-defined missing values for dependent variables are treated as missing. |
| | Cases Used | Statistics are based on cases with no missing values for any dependent variable or factor used. |
| Syntax | | EXAMINE VARIABLES=cap_func lim_fisico dor lim_emocional estado_geral vitalidade asp_sociais saude_mental<br>/PLOT NONE<br>/STATISTICS DESCRIPTIVES<br>/CINTERVAL 95<br>/MISSING LISTWISE<br>/NOTOTAL. |
| Resources | Processor Time | 00:00:00,00 |
| | Elapsed Time | 00:00:00,01 |

```
[InsiemeDati1] \\Mac\iCloud\Lavoro\FMABC\Orientações\Orientações\finite\Marin
a Rodrigues - Caio e Fabia\Marina Farias\data_article_6.sav
```

**Case Processing Summary**

| Grau_Endometriodo_Cat | | Cases | | | | | |
|---|---|---|---|---|---|---|---|
| | | Valid | | Missing | | Total | |
| | | N | Percent | N | Percent | N | Percent |
| Grau I/II | cap_func | 26 | 100,0% | 0 | 0,0% | 26 | 100,0% |
| | lim_fisico | 26 | 100,0% | 0 | 0,0% | 26 | 100,0% |
| | dor | 26 | 100,0% | 0 | 0,0% | 26 | 100,0% |
| | lim_emocional | 26 | 100,0% | 0 | 0,0% | 26 | 100,0% |
| | estado_geral | 26 | 100,0% | 0 | 0,0% | 26 | 100,0% |
| | vitalidade | 26 | 100,0% | 0 | 0,0% | 26 | 100,0% |
| | asp_sociais | 26 | 100,0% | 0 | 0,0% | 26 | 100,0% |
| | saude_mental | 26 | 100,0% | 0 | 0,0% | 26 | 100,0% |
| Grau III/IV | cap_func | 80 | 100,0% | 0 | 0,0% | 80 | 100,0% |
| | lim_fisico | 80 | 100,0% | 0 | 0,0% | 80 | 100,0% |
| | dor | 80 | 100,0% | 0 | 0,0% | 80 | 100,0% |
| | lim_emocional | 80 | 100,0% | 0 | 0,0% | 80 | 100,0% |
| | estado_geral | 80 | 100,0% | 0 | 0,0% | 80 | 100,0% |
| | vitalidade | 80 | 100,0% | 0 | 0,0% | 80 | 100,0% |
| | asp_sociais | 80 | 100,0% | 0 | 0,0% | 80 | 100,0% |
| | saude_mental | 80 | 100,0% | 0 | 0,0% | 80 | 100,0% |

**Descriptives**

| Grau_Endometriodo_Cat | | | | Statistic |
|---|---|---|---|---|
| Grau I/II | cap_func | Mean | | 81,35 |
| | | 95% Confidence Interval for Mean | Lower Bound | 73,80 |
| | | | Upper Bound | 88,89 |
| | | 5% Trimmed Mean | | 82,97 |
| | | Median | | 87,50 |
| | | Variance | | 349,115 |
| | | Std. Deviation | | 18,685 |
| | | Minimum | | 30 |
| | | Maximum | | 100 |
| | | Range | | 70 |
| | | Interquartile Range | | 25 |
| | | Skewness | | -1,111 |
| | | Kurtosis | | ,853 |
| | lim_fisico | Mean | | 71,15 |
| | | 95% Confidence Interval for Mean | Lower Bound | 55,59 |
| | | | Upper Bound | 86,72 |
| | | 5% Trimmed Mean | | 73,50 |
| | | Median | | 100,00 |
| | | Variance | | 1484,615 |

**Descriptives**

| Grau_Endometriodo_Cat | | | | Std. Error |
|---|---|---|---|---|
| Grau I/II | cap_func | Mean | | 3,664 |
| | | 95% Confidence Interval for Mean | Lower Bound | |
| | | | Upper Bound | |
| | | 5% Trimmed Mean | | |
| | | Median | | |
| | | Variance | | |
| | | Std. Deviation | | |
| | | Minimum | | |
| | | Maximum | | |
| | | Range | | |
| | | Interquartile Range | | |
| | | Skewness | | ,456 |
| | | Kurtosis | | ,887 |
| | lim_fisico | Mean | | 7,556 |
| | | 95% Confidence Interval for Mean | Lower Bound | |
| | | | Upper Bound | |
| | | 5% Trimmed Mean | | |
| | | Median | | |
| | | Variance | | |

**Descriptives**

| Grau_Endometriodo_Cat | | | | Statistic |
|---|---|---|---|---|
| | | Std. Deviation | | 38,531 |
| | | Minimum | | 0 |
| | | Maximum | | 100 |
| | | Range | | 100 |
| | | Interquartile Range | | 56 |
| | | Skewness | | -1,000 |
| | | Kurtosis | | -,588 |
| | dor | Mean | | 67,307692 |
| | | 95% Confidence Interval for Mean | Lower Bound | 57,106285 |
| | | | Upper Bound | 77,509099 |
| | | 5% Trimmed Mean | | 67,508547 |
| | | Median | | 73,000000 |
| | | Variance | | 637,902 |
| | | Std. Deviation | | 25,2567127 |
| | | Minimum | | 31,0000 |
| | | Maximum | | 100,0000 |
| | | Range | | 69,0000 |
| | | Interquartile Range | | 43,0000 |
| | | Skewness | | -,133 |
| | | Kurtosis | | -1,527 |
| | lim_emocional | Mean | | 53,846154 |
| | | 95% Confidence Interval for Mean | Lower Bound | 34,399935 |
| | | | Upper Bound | 73,292372 |
| | | 5% Trimmed Mean | | 54,273504 |
| | | Median | | 66,666667 |
| | | Variance | | 2317,949 |
| | | Std. Deviation | | 48,1450799 |
| | | Minimum | | ,0000 |
| | | Maximum | | 100,0000 |
| | | Range | | 100,0000 |
| | | Interquartile Range | | 100,0000 |
| | | Skewness | | -,120 |
| | | Kurtosis | | -2,037 |
| | estado_geral | Mean | | 58,692308 |
| | | 95% Confidence Interval for Mean | Lower Bound | 52,002750 |
| | | | Upper Bound | 65,381865 |
| | | 5% Trimmed Mean | | 59,017094 |
| | | Median | | 62,000000 |
| | | Variance | | 274,302 |

**Descriptives**

| Grau_Endometriodo_Cat | | | | Std. Error |
|---|---|---|---|---|
| | | Std. Deviation | | |
| | | Minimum | | |
| | | Maximum | | |
| | | Range | | |
| | | Interquartile Range | | |
| | | Skewness | | ,456 |
| | | Kurtosis | | ,887 |
| | dor | Mean | | 4,9532489 |
| | | 95% Confidence Interval for Mean | Lower Bound | |
| | | | Upper Bound | |
| | | 5% Trimmed Mean | | |
| | | Median | | |
| | | Variance | | |
| | | Std. Deviation | | |
| | | Minimum | | |
| | | Maximum | | |
| | | Range | | |
| | | Interquartile Range | | |
| | | Skewness | | ,456 |
| | | Kurtosis | | ,887 |
| | lim_emocional | Mean | | 9,4420270 |
| | | 95% Confidence Interval for Mean | Lower Bound | |
| | | | Upper Bound | |
| | | 5% Trimmed Mean | | |
| | | Median | | |
| | | Variance | | |
| | | Std. Deviation | | |
| | | Minimum | | |
| | | Maximum | | |
| | | Range | | |
| | | Interquartile Range | | |
| | | Skewness | | ,456 |
| | | Kurtosis | | ,887 |
| | estado_geral | Mean | | 3,2480855 |
| | | 95% Confidence Interval for Mean | Lower Bound | |
| | | | Upper Bound | |
| | | 5% Trimmed Mean | | |
| | | Median | | |
| | | Variance | | |

**Descriptives**

| Grau_Endometriodo_Cat | | | | Statistic |
|---|---|---|---|---|
| | | Std. Deviation | | 16,5620512 |
| | | Minimum | | 25,0000 |
| | | Maximum | | 87,0000 |
| | | Range | | 62,0000 |
| | | Interquartile Range | | 25,0000 |
| | | Skewness | | -,358 |
| | | Kurtosis | | -,358 |
| | vitalidade | Mean | | 54,42 |
| | | 95% Confidence Interval for Mean | Lower Bound | 48,48 |
| | | | Upper Bound | 60,37 |
| | | 5% Trimmed Mean | | 54,57 |
| | | Median | | 55,00 |
| | | Variance | | 216,654 |
| | | Std. Deviation | | 14,719 |
| | | Minimum | | 25 |
| | | Maximum | | 80 |
| | | Range | | 55 |
| | | Interquartile Range | | 26 |
| | | Skewness | | -,071 |
| | | Kurtosis | | -,877 |
| | asp_sociais | Mean | | 66,346 |
| | | 95% Confidence Interval for Mean | Lower Bound | 55,682 |
| | | | Upper Bound | 77,011 |
| | | 5% Trimmed Mean | | 67,308 |
| | | Median | | 68,750 |
| | | Variance | | 697,115 |
| | | Std. Deviation | | 26,4029 |
| | | Minimum | | 12,5 |
| | | Maximum | | 100,0 |
| | | Range | | 87,5 |
| | | Interquartile Range | | 40,6 |
| | | Skewness | | -,357 |
| | | Kurtosis | | -,907 |
| | saude_mental | Mean | | 59,54 |
| | | 95% Confidence Interval for Mean | Lower Bound | 50,99 |
| | | | Upper Bound | 68,09 |
| | | 5% Trimmed Mean | | 59,04 |
| | | Median | | 60,00 |
| | | Variance | | 448,418 |

**Descriptives**

| Grau_Endometriodo_Cat | | | | Std. Error |
| --- | --- | --- | --- | --- |
| | | Std. Deviation | | |
| | | Minimum | | |
| | | Maximum | | |
| | | Range | | |
| | | Interquartile Range | | |
| | | Skewness | | ,456 |
| | | Kurtosis | | ,887 |
| | vitalidade | Mean | | 2,887 |
| | | 95% Confidence Interval for Mean | Lower Bound | |
| | | | Upper Bound | |
| | | 5% Trimmed Mean | | |
| | | Median | | |
| | | Variance | | |
| | | Std. Deviation | | |
| | | Minimum | | |
| | | Maximum | | |
| | | Range | | |
| | | Interquartile Range | | |
| | | Skewness | | ,456 |
| | | Kurtosis | | ,887 |
| | asp_sociais | Mean | | 5,1780 |
| | | 95% Confidence Interval for Mean | Lower Bound | |
| | | | Upper Bound | |
| | | 5% Trimmed Mean | | |
| | | Median | | |
| | | Variance | | |
| | | Std. Deviation | | |
| | | Minimum | | |
| | | Maximum | | |
| | | Range | | |
| | | Interquartile Range | | |
| | | Skewness | | ,456 |
| | | Kurtosis | | ,887 |
| | saude_mental | Mean | | 4,153 |
| | | 95% Confidence Interval for Mean | Lower Bound | |
| | | | Upper Bound | |
| | | 5% Trimmed Mean | | |
| | | Median | | |
| | | Variance | | |

**Descriptives**

| Grau_Endometriodo_Cat | | | | Statistic |
|---|---|---|---|---|
| | | Std. Deviation | | 21,176 |
| | | Minimum | | 28 |
| | | Maximum | | 100 |
| | | Range | | 72 |
| | | Interquartile Range | | 38 |
| | | Skewness | | ,194 |
| | | Kurtosis | | -1,248 |
| Grau III/IV | cap_func | Mean | | 82,20 |
| | | 95% Confidence Interval for Mean | Lower Bound | 77,94 |
| | | | Upper Bound | 86,46 |
| | | 5% Trimmed Mean | | 84,11 |
| | | Median | | 85,00 |
| | | Variance | | 366,491 |
| | | Std. Deviation | | 19,144 |
| | | Minimum | | 10 |
| | | Maximum | | 100 |
| | | Range | | 90 |
| | | Interquartile Range | | 28 |
| | | Skewness | | -1,400 |
| | | Kurtosis | | 1,959 |
| | lim_fisico | Mean | | 72,24 |
| | | 95% Confidence Interval for Mean | Lower Bound | 63,53 |
| | | | Upper Bound | 80,95 |
| | | 5% Trimmed Mean | | 74,71 |
| | | Median | | 100,00 |
| | | Variance | | 1531,272 |
| | | Std. Deviation | | 39,131 |
| | | Minimum | | 0 |
| | | Maximum | | 100 |
| | | Range | | 100 |
| | | Interquartile Range | | 69 |
| | | Skewness | | -1,014 |
| | | Kurtosis | | -,688 |
| | dor | Mean | | 60,900000 |
| | | 95% Confidence Interval for Mean | Lower Bound | 55,245203 |
| | | | Upper Bound | 66,554797 |
| | | 5% Trimmed Mean | | 61,277778 |
| | | Median | | 57,000000 |
| | | Variance | | 645,686 |

**Descriptives**

| Grau_Endometriodo_Cat | | | | Std. Error |
|---|---|---|---|---|
| | | Std. Deviation | | |
| | | Minimum | | |
| | | Maximum | | |
| | | Range | | |
| | | Interquartile Range | | |
| | | Skewness | | ,456 |
| | | Kurtosis | | ,887 |
| Grau III/IV | cap_func | Mean | | 2,140 |
| | | 95% Confidence Interval for Mean | Lower Bound | |
| | | | Upper Bound | |
| | | 5% Trimmed Mean | | |
| | | Median | | |
| | | Variance | | |
| | | Std. Deviation | | |
| | | Minimum | | |
| | | Maximum | | |
| | | Range | | |
| | | Interquartile Range | | |
| | | Skewness | | ,269 |
| | | Kurtosis | | ,532 |
| | lim_fisico | Mean | | 4,375 |
| | | 95% Confidence Interval for Mean | Lower Bound | |
| | | | Upper Bound | |
| | | 5% Trimmed Mean | | |
| | | Median | | |
| | | Variance | | |
| | | Std. Deviation | | |
| | | Minimum | | |
| | | Maximum | | |
| | | Range | | |
| | | Interquartile Range | | |
| | | Skewness | | ,269 |
| | | Kurtosis | | ,532 |
| | dor | Mean | | 2,8409639 |
| | | 95% Confidence Interval for Mean | Lower Bound | |
| | | | Upper Bound | |
| | | 5% Trimmed Mean | | |
| | | Median | | |
| | | Variance | | |

**Descriptives**

| Grau_Endometriodo_Cat | | | | Statistic |
|---|---|---|---|---|
| | | Std. Deviation | | 25,4103537 |
| | | Minimum | | 10,0000 |
| | | Maximum | | 100,0000 |
| | | Range | | 90,0000 |
| | | Interquartile Range | | 43,0000 |
| | | Skewness | | ,113 |
| | | Kurtosis | | -,965 |
| | lim_emocional | Mean | | 65,795833 |
| | | 95% Confidence Interval for Mean | Lower Bound | 57,369015 |
| | | | Upper Bound | 74,222652 |
| | | 5% Trimmed Mean | | 67,550926 |
| | | Median | | 66,666667 |
| | | Variance | | 1433,886 |
| | | Std. Deviation | | 37,8666880 |
| | | Minimum | | ,0000 |
| | | Maximum | | 100,0000 |
| | | Range | | 100,0000 |
| | | Interquartile Range | | 66,6667 |
| | | Skewness | | -,638 |
| | | Kurtosis | | -1,067 |
| | estado_geral | Mean | | 60,537500 |
| | | 95% Confidence Interval for Mean | Lower Bound | 56,626510 |
| | | | Upper Bound | 64,448490 |
| | | 5% Trimmed Mean | | 60,500000 |
| | | Median | | 57,000000 |
| | | Variance | | 308,859 |
| | | Std. Deviation | | 17,5743943 |
| | | Minimum | | 22,0000 |
| | | Maximum | | 97,0000 |
| | | Range | | 75,0000 |
| | | Interquartile Range | | 20,0000 |
| | | Skewness | | ,126 |
| | | Kurtosis | | -,034 |
| | vitalidade | Mean | | 56,24 |
| | | 95% Confidence Interval for Mean | Lower Bound | 53,71 |
| | | | Upper Bound | 58,77 |
| | | 5% Trimmed Mean | | 56,51 |
| | | Median | | 55,00 |
| | | Variance | | 129,500 |

**Descriptives**

| Grau_Endometriodo_Cat | | | | Std. Error |
|---|---|---|---|---|
| | | Std. Deviation | | |
| | | Minimum | | |
| | | Maximum | | |
| | | Range | | |
| | | Interquartile Range | | |
| | | Skewness | | ,269 |
| | | Kurtosis | | ,532 |
| | lim_emocional | Mean | | 4,2336244 |
| | | 95% Confidence Interval for Mean | Lower Bound | |
| | | | Upper Bound | |
| | | 5% Trimmed Mean | | |
| | | Median | | |
| | | Variance | | |
| | | Std. Deviation | | |
| | | Minimum | | |
| | | Maximum | | |
| | | Range | | |
| | | Interquartile Range | | |
| | | Skewness | | ,269 |
| | | Kurtosis | | ,532 |
| | estado_geral | Mean | | 1,9648770 |
| | | 95% Confidence Interval for Mean | Lower Bound | |
| | | | Upper Bound | |
| | | 5% Trimmed Mean | | |
| | | Median | | |
| | | Variance | | |
| | | Std. Deviation | | |
| | | Minimum | | |
| | | Maximum | | |
| | | Range | | |
| | | Interquartile Range | | |
| | | Skewness | | ,269 |
| | | Kurtosis | | ,532 |
| | vitalidade | Mean | | 1,272 |
| | | 95% Confidence Interval for Mean | Lower Bound | |
| | | | Upper Bound | |
| | | 5% Trimmed Mean | | |
| | | Median | | |
| | | Variance | | |

**Descriptives**

| Grau_Endometriodo_Cat | | | | Statistic |
|---|---|---|---|---|
| | | Std. Deviation | | 11,380 |
| | | Minimum | | 25 |
| | | Maximum | | 75 |
| | | Range | | 50 |
| | | Interquartile Range | | 20 |
| | | Skewness | | -,354 |
| | | Kurtosis | | -,574 |
| | asp_sociais | Mean | | 66,200 |
| | | 95% Confidence Interval for Mean | Lower Bound | 60,953 |
| | | | Upper Bound | 71,447 |
| | | 5% Trimmed Mean | | 66,958 |
| | | Median | | 62,500 |
| | | Variance | | 555,928 |
| | | Std. Deviation | | 23,5781 |
| | | Minimum | | 12,5 |
| | | Maximum | | 100,0 |
| | | Range | | 87,5 |
| | | Interquartile Range | | 37,5 |
| | | Skewness | | -,150 |
| | | Kurtosis | | -,705 |
| | saude_mental | Mean | | 59,23 |
| | | 95% Confidence Interval for Mean | Lower Bound | 55,10 |
| | | | Upper Bound | 63,35 |
| | | 5% Trimmed Mean | | 59,08 |
| | | Median | | 59,50 |
| | | Variance | | 343,164 |
| | | Std. Deviation | | 18,525 |
| | | Minimum | | 16 |
| | | Maximum | | 100 |
| | | Range | | 84 |
| | | Interquartile Range | | 31 |
| | | Skewness | | ,123 |
| | | Kurtosis | | -,443 |

**Descriptives**

| Grau_Endometriodo_Cat | | | | Std. Error |
|---|---|---|---|---|
| | | Std. Deviation | | |
| | | Minimum | | |
| | | Maximum | | |
| | | Range | | |
| | | Interquartile Range | | |
| | | Skewness | | ,269 |
| | | Kurtosis | | ,532 |
| | asp_sociais | Mean | | 2,6361 |
| | | 95% Confidence Interval for Mean | Lower Bound | |
| | | | Upper Bound | |
| | | 5% Trimmed Mean | | |
| | | Median | | |
| | | Variance | | |
| | | Std. Deviation | | |
| | | Minimum | | |
| | | Maximum | | |
| | | Range | | |
| | | Interquartile Range | | |
| | | Skewness | | ,269 |
| | | Kurtosis | | ,532 |
| | saude_mental | Mean | | 2,071 |
| | | 95% Confidence Interval for Mean | Lower Bound | |
| | | | Upper Bound | |
| | | 5% Trimmed Mean | | |
| | | Median | | |
| | | Variance | | |
| | | Std. Deviation | | |
| | | Minimum | | |
| | | Maximum | | |
| | | Range | | |
| | | Interquartile Range | | |
| | | Skewness | | ,269 |
| | | Kurtosis | | ,532 |

```
 SPLIT FILE OFF.
CORRELATIONS
  /VARIABLES=idade tempo_ACO estado_geral vitalidade asp_sociais saude_mental
  /PRINT=TWOTAIL NOSIG
  /MISSING=PAIRWISE.
```

## Correlations

**Notes**

| | | |
|---|---|---|
| Output Created | | 08-MAY-2020 11:34:54 |
| Comments | | |
| Input | Data | \\Mac\iCloud\Lavoro\FMABC\Orientações\Orientações\finite\Marina Rodrigues - Caio e Fabia\Marina Farias\data_article_6.sav |
| | Active Dataset | InsiemeDati1 |
| | Filter | <none> |
| | Weight | <none> |
| | Split File | <none> |
| | N of Rows in Working Data File | 106 |
| Missing Value Handling | Definition of Missing | User-defined missing values are treated as missing. |
| | Cases Used | Statistics for each pair of variables are based on all the cases with valid data for that pair. |
| Syntax | | CORRELATIONS /VARIABLES=idade tempo_ACO estado_geral vitalidade asp_sociais saude_mental /PRINT=TWOTAIL NOSIG /MISSING=PAIRWISE. |
| Resources | Processor Time | 00:00:00,00 |
| | Elapsed Time | 00:00:00,01 |

[InsiemeDati1] \\Mac\iCloud\Lavoro\FMABC\Orientações\Orientações\finite\Marina Rodrigues - Caio e Fabia\Marina Farias\data_article_6.sav

**Correlations**

| | | idade | tempo_ACO | estado_geral | vitalidade |
|---|---|---|---|---|---|
| idade | Pearson Correlation | 1 | -,007 | ,177 | ,006 |
| | Sig. (2-tailed) | | ,942 | ,069 | ,951 |
| | N | 106 | 106 | 106 | 106 |
| tempo_ACO | Pearson Correlation | -,007 | 1 | ,084 | ,095 |
| | Sig. (2-tailed) | ,942 | | ,390 | ,332 |
| | N | 106 | 106 | 106 | 106 |
| estado_geral | Pearson Correlation | ,177 | ,084 | 1 | ,420** |
| | Sig. (2-tailed) | ,069 | ,390 | | ,000 |
| | N | 106 | 106 | 106 | 106 |
| vitalidade | Pearson Correlation | ,006 | ,095 | ,420** | 1 |
| | Sig. (2-tailed) | ,951 | ,332 | ,000 | |
| | N | 106 | 106 | 106 | 106 |
| asp_sociais | Pearson Correlation | ,084 | ,077 | ,526** | ,447** |
| | Sig. (2-tailed) | ,394 | ,430 | ,000 | ,000 |
| | N | 106 | 106 | 106 | 106 |
| saude_mental | Pearson Correlation | ,009 | ,054 | ,465** | ,629** |
| | Sig. (2-tailed) | ,928 | ,583 | ,000 | ,000 |
| | N | 106 | 106 | 106 | 106 |

**Correlations**

| | | asp_sociais | saude_mental |
|---|---|---|---|
| idade | Pearson Correlation | ,084 | ,009 |
| | Sig. (2-tailed) | ,394 | ,928 |
| | N | 106 | 106 |
| tempo_ACO | Pearson Correlation | ,077 | ,054 |
| | Sig. (2-tailed) | ,430 | ,583 |
| | N | 106 | 106 |
| estado_geral | Pearson Correlation | ,526** | ,465** |
| | Sig. (2-tailed) | ,000 | ,000 |
| | N | 106 | 106 |
| vitalidade | Pearson Correlation | ,447** | ,629** |
| | Sig. (2-tailed) | ,000 | ,000 |
| | N | 106 | 106 |
| asp_sociais | Pearson Correlation | 1 | ,532** |
| | Sig. (2-tailed) | | ,000 |
| | N | 106 | 106 |
| saude_mental | Pearson Correlation | ,532** | 1 |
| | Sig. (2-tailed) | ,000 | |
| | N | 106 | 106 |

**. Correlation is significant at the 0.01 level (2-tailed).

```
NONPAR CORR
  /VARIABLES=temp_infert menarca estado_geral vitalidade asp_sociais saude_mental
  /PRINT=SPEARMAN TWOTAIL NOSIG
  /MISSING=PAIRWISE.
```

# Nonparametric Correlations

**Notes**

| | | |
|---|---|---|
| Output Created | | 08-MAY-2020 11:35:19 |
| Comments | | |
| Input | Data | \\Mac\iCloud\Lavoro\FMABC\Orientações\Orientações\finite\Marina Rodrigues - Caio e Fabia\Marina Farias\data_article_6.sav |
| | Active Dataset | InsiemeDati1 |
| | Filter | <none> |
| | Weight | <none> |
| | Split File | <none> |
| | N of Rows in Working Data File | 106 |
| Missing Value Handling | Definition of Missing | User-defined missing values are treated as missing. |
| | Cases Used | Statistics for each pair of variables are based on all the cases with valid data for that pair. |
| Syntax | | NONPAR CORR<br>/VARIABLES=temp_infert menarca estado_geral vitalidade asp_sociais saude_mental<br>/PRINT=SPEARMAN TWOTAIL NOSIG<br>/MISSING=PAIRWISE. |
| Resources | Processor Time | 00:00:00,00 |
| | Elapsed Time | 00:00:00,01 |
| | Number of Cases Allowed | 92521 cases[a] |

a. Based on availability of workspace memory

```
[InsiemeDati1] \\Mac\iCloud\Lavoro\FMABC\Orientações\Orientações\finite\Marin
a Rodrigues - Caio e Fabia\Marina Farias\data_article_6.sav
```

**Correlations**

| | | | temp_infert | menarca | estado_geral |
|---|---|---|---|---|---|
| Spearman's rho | temp_infert | Correlation Coefficient | 1,000 | -,046 | ,024 |
| | | Sig. (2-tailed) | . | ,642 | ,804 |
| | | N | 106 | 106 | 106 |
| | menarca | Correlation Coefficient | -,046 | 1,000 | ,064 |
| | | Sig. (2-tailed) | ,642 | . | ,512 |
| | | N | 106 | 106 | 106 |
| | estado_geral | Correlation Coefficient | ,024 | ,064 | 1,000 |
| | | Sig. (2-tailed) | ,804 | ,512 | . |
| | | N | 106 | 106 | 106 |
| | vitalidade | Correlation Coefficient | ,022 | ,101 | ,416** |
| | | Sig. (2-tailed) | ,823 | ,302 | ,000 |
| | | N | 106 | 106 | 106 |
| | asp_sociais | Correlation Coefficient | ,028 | ,047 | ,485** |
| | | Sig. (2-tailed) | ,778 | ,631 | ,000 |
| | | N | 106 | 106 | 106 |
| | saude_mental | Correlation Coefficient | ,030 | ,007 | ,450** |
| | | Sig. (2-tailed) | ,759 | ,945 | ,000 |
| | | N | 106 | 106 | 106 |

**Correlations**

| | | | vitalidade | asp_sociais | saude_mental |
|---|---|---|---|---|---|
| Spearman's rho | temp_infert | Correlation Coefficient | ,022 | ,028 | ,030 |
| | | Sig. (2-tailed) | ,823 | ,778 | ,759 |
| | | N | 106 | 106 | 106 |
| | menarca | Correlation Coefficient | ,101 | ,047 | ,007 |
| | | Sig. (2-tailed) | ,302 | ,631 | ,945 |
| | | N | 106 | 106 | 106 |
| | estado_geral | Correlation Coefficient | ,416** | ,485** | ,450** |
| | | Sig. (2-tailed) | ,000 | ,000 | ,000 |
| | | N | 106 | 106 | 106 |
| | vitalidade | Correlation Coefficient | 1,000 | ,439** | ,610** |
| | | Sig. (2-tailed) | . | ,000 | ,000 |
| | | N | 106 | 106 | 106 |
| | asp_sociais | Correlation Coefficient | ,439** | 1,000 | ,532** |
| | | Sig. (2-tailed) | ,000 | . | ,000 |
| | | N | 106 | 106 | 106 |
| | saude_mental | Correlation Coefficient | ,610** | ,532** | 1,000 |
| | | Sig. (2-tailed) | ,000 | ,000 | . |
| | | N | 106 | 106 | 106 |

**. Correlation is significant at the 0.01 level (2-tailed).

```
NONPAR CORR
  /VARIABLES=idade temp_infert menarca tempo_ACO cap_func lim_fisico dor lim_emocional
  /PRINT=SPEARMAN TWOTAIL NOSIG
  /MISSING=PAIRWISE.
```

# Nonparametric Correlations

**Notes**

| | | |
|---|---|---|
| Output Created | | 08-MAY-2020 11:35:52 |
| Comments | | |
| Input | Data | \\Mac\iCloud\Lavoro\FMABC\Orientações\Orientações\finite\Marina Rodrigues - Caio e Fabia\Marina Farias\data_article_6.sav |
| | Active Dataset | InsiemeDati1 |
| | Filter | <none> |
| | Weight | <none> |
| | Split File | <none> |
| | N of Rows in Working Data File | 106 |
| Missing Value Handling | Definition of Missing | User-defined missing values are treated as missing. |
| | Cases Used | Statistics for each pair of variables are based on all the cases with valid data for that pair. |
| Syntax | | NONPAR CORR /VARIABLES=idade temp_infert menarca tempo_ACO cap_func lim_fisico dor lim_emocional /PRINT=SPEARMAN TWOTAIL NOSIG /MISSING=PAIRWISE. |
| Resources | Processor Time | 00:00:00,00 |
| | Elapsed Time | 00:00:00,04 |
| | Number of Cases Allowed | 74898 cases[a] |

a. Based on availability of workspace memory

```
[InsiemeDati1] \\Mac\iCloud\Lavoro\FMABC\Orientações\Orientações\finite\Marin
a Rodrigues - Caio e Fabia\Marina Farias\data_article_6.sav
```

**Correlations**

| | | | idade | temp_infert | menarca |
|---|---|---|---|---|---|
| Spearman's rho | idade | Correlation Coefficient | 1,000 | ,088 | -,026 |
| | | Sig. (2-tailed) | . | ,372 | ,790 |
| | | N | 106 | 106 | 106 |
| | temp_infert | Correlation Coefficient | ,088 | 1,000 | -,046 |
| | | Sig. (2-tailed) | ,372 | . | ,642 |
| | | N | 106 | 106 | 106 |
| | menarca | Correlation Coefficient | -,026 | -,046 | 1,000 |
| | | Sig. (2-tailed) | ,790 | ,642 | . |
| | | N | 106 | 106 | 106 |
| | tempo_ACO | Correlation Coefficient | -,041 | -,144 | -,023 |
| | | Sig. (2-tailed) | ,676 | ,141 | ,817 |
| | | N | 106 | 106 | 106 |
| | cap_func | Correlation Coefficient | ,114 | ,108 | ,069 |
| | | Sig. (2-tailed) | ,245 | ,271 | ,481 |
| | | N | 106 | 106 | 106 |
| | lim_fisico | Correlation Coefficient | ,000 | -,044 | ,152 |
| | | Sig. (2-tailed) | ,998 | ,653 | ,120 |
| | | N | 106 | 106 | 106 |
| | dor | Correlation Coefficient | ,072 | -,030 | ,057 |
| | | Sig. (2-tailed) | ,463 | ,760 | ,562 |
| | | N | 106 | 106 | 106 |
| | lim_emocional | Correlation Coefficient | ,016 | ,173 | ,006 |
| | | Sig. (2-tailed) | ,868 | ,077 | ,954 |
| | | N | 106 | 106 | 106 |

**Correlations**

| | | | tempo_ACO | cap_func | lim_fisico |
|---|---|---|---|---|---|
| Spearman's rho | idade | Correlation Coefficient | -,041 | ,114 | ,000 |
| | | Sig. (2-tailed) | ,676 | ,245 | ,998 |
| | | N | 106 | 106 | 106 |
| | temp_infert | Correlation Coefficient | -,144 | ,108 | -,044 |
| | | Sig. (2-tailed) | ,141 | ,271 | ,653 |
| | | N | 106 | 106 | 106 |
| | menarca | Correlation Coefficient | -,023 | ,069 | ,152 |
| | | Sig. (2-tailed) | ,817 | ,481 | ,120 |
| | | N | 106 | 106 | 106 |
| | tempo_ACO | Correlation Coefficient | 1,000 | -,002 | ,082 |
| | | Sig. (2-tailed) | . | ,985 | ,404 |
| | | N | 106 | 106 | 106 |
| | cap_func | Correlation Coefficient | -,002 | 1,000 | ,436** |
| | | Sig. (2-tailed) | ,985 | . | ,000 |
| | | N | 106 | 106 | 106 |
| | lim_fisico | Correlation Coefficient | ,082 | ,436** | 1,000 |
| | | Sig. (2-tailed) | ,404 | ,000 | . |
| | | N | 106 | 106 | 106 |
| | dor | Correlation Coefficient | ,031 | ,303** | ,339** |
| | | Sig. (2-tailed) | ,750 | ,002 | ,000 |
| | | N | 106 | 106 | 106 |
| | lim_emocional | Correlation Coefficient | ,070 | ,385** | ,493** |
| | | Sig. (2-tailed) | ,474 | ,000 | ,000 |
| | | N | 106 | 106 | 106 |

**Correlations**

| | | | dor | lim_emocional |
|---|---|---|---|---|
| Spearman's rho | idade | Correlation Coefficient | ,072 | ,016 |
| | | Sig. (2-tailed) | ,463 | ,868 |
| | | N | 106 | 106 |
| | temp_infert | Correlation Coefficient | -,030 | ,173 |
| | | Sig. (2-tailed) | ,760 | ,077 |
| | | N | 106 | 106 |
| | menarca | Correlation Coefficient | ,057 | ,006 |
| | | Sig. (2-tailed) | ,562 | ,954 |
| | | N | 106 | 106 |
| | tempo_ACO | Correlation Coefficient | ,031 | ,070 |
| | | Sig. (2-tailed) | ,750 | ,474 |
| | | N | 106 | 106 |
| | cap_func | Correlation Coefficient | ,303** | ,385** |
| | | Sig. (2-tailed) | ,002 | ,000 |
| | | N | 106 | 106 |
| | lim_fisico | Correlation Coefficient | ,339** | ,493** |
| | | Sig. (2-tailed) | ,000 | ,000 |
| | | N | 106 | 106 |
| | dor | Correlation Coefficient | 1,000 | ,186 |
| | | Sig. (2-tailed) | . | ,056 |
| | | N | 106 | 106 |
| | lim_emocional | Correlation Coefficient | ,186 | 1,000 |
| | | Sig. (2-tailed) | ,056 | . |
| | | N | 106 | 106 |

**. Correlation is significant at the 0.01 level (2-tailed).

```
T-TEST GROUPS=tipo_infert(0 1)
 /MISSING=ANALYSIS
 /VARIABLES=estado_geral vitalidade asp_sociais saude_mental
 /CRITERIA=CI(.95).
```

# T-Test

**Notes**

| | | |
|---|---|---|
| Output Created | | 08-MAY-2020 11:42:26 |
| Comments | | |
| Input | Data | \\Mac\iCloud\Lavoro\FMABC\Orientações\Orientações\finite\Marina Rodrigues - Caio e Fabia\Marina Farias\data_article_6.sav |
| | Active Dataset | InsiemeDati1 |
| | Filter | <none> |
| | Weight | <none> |
| | Split File | <none> |
| | N of Rows in Working Data File | 106 |
| Missing Value Handling | Definition of Missing | User defined missing values are treated as missing. |
| | Cases Used | Statistics for each analysis are based on the cases with no missing or out-of-range data for any variable in the analysis. |
| Syntax | | T-TEST GROUPS=tipo_infert(0 1)<br>/MISSING=ANALYSIS<br>/VARIABLES=estado_geral vitalidade asp_sociais saude_mental<br>/CRITERIA=CI(.95). |
| Resources | Processor Time | 00:00:00,00 |
| | Elapsed Time | 00:00:00,01 |

```
[InsiemeDati1] \\Mac\iCloud\Lavoro\FMABC\Orientações\Orientações\finite\Marin
a Rodrigues - Caio e Fabia\Marina Farias\data_article_6.sav
```

**Group Statistics**

| | tipo_infert | N | Mean | Std. Deviation | Std. Error Mean |
|---|---|---|---|---|---|
| estado_geral | Primária | 92 | 60,358696 | 17,7098604 | 1,8463806 |
| | Secundária | 14 | 58,285714 | 14,4936515 | 3,8735913 |
| vitalidade | Primária | 92 | 55,96 | 12,587 | 1,312 |
| | Secundária | 14 | 54,71 | 9,903 | 2,647 |
| asp_sociais | Primária | 92 | 66,842 | 24,9624 | 2,6025 |
| | Secundária | 14 | 62,250 | 18,3688 | 4,9093 |
| saude_mental | Primária | 92 | 58,83 | 19,860 | 2,071 |
| | Secundária | 14 | 62,43 | 13,224 | 3,534 |

**Independent Samples Test**

| | | Levene's Test for Equality of Variances | | t-test for Equality of Means | |
|---|---|---|---|---|---|
| | | F | Sig. | t | df |
| estado_geral | Equal variances assumed | 1,386 | ,242 | ,417 | 104 |
| | Equal variances not assumed | | | ,483 | 19,435 |
| vitalidade | Equal variances assumed | 1,122 | ,292 | ,353 | 104 |
| | Equal variances not assumed | | | ,421 | 20,005 |
| asp_sociais | Equal variances assumed | 3,045 | ,084 | ,661 | 104 |
| | Equal variances not assumed | | | ,826 | 21,095 |
| saude_mental | Equal variances assumed | 3,438 | ,067 | -,656 | 104 |
| | Equal variances not assumed | | | -,879 | 23,066 |

**Independent Samples Test**

| | | t-test for Equality of Means | | |
|---|---|---|---|---|
| | | Sig. (2-tailed) | Mean Difference | Std. Error Difference |
| estado_geral | Equal variances assumed | ,678 | 2,0729814 | 4,9745786 |
| | Equal variances not assumed | ,634 | 2,0729814 | 4,2911340 |
| vitalidade | Equal variances assumed | ,725 | 1,242 | 3,524 |
| | Equal variances not assumed | ,679 | 1,242 | 2,954 |
| asp_sociais | Equal variances assumed | ,510 | 4,5924 | 6,9529 |
| | Equal variances not assumed | ,418 | 4,5924 | 5,5564 |
| saude_mental | Equal variances assumed | ,514 | -3,602 | 5,496 |
| | Equal variances not assumed | ,388 | -3,602 | 4,096 |

**Independent Samples Test**

| | | t-test for Equality of Means | |
|---|---|---|---|
| | | 95% Confidence Interval of the Difference | |
| | | Lower | Upper |
| estado_geral | Equal variances assumed | -7,7917947 | 11,9377575 |
| | Equal variances not assumed | -6,8948773 | 11,0408401 |
| vitalidade | Equal variances assumed | -5,746 | 8,230 |
| | Equal variances not assumed | -4,920 | 7,404 |
| asp_sociais | Equal variances assumed | -9,1954 | 18,3802 |
| | Equal variances not assumed | -6,9597 | 16,1445 |
| saude_mental | Equal variances assumed | -14,500 | 7,295 |
| | Equal variances not assumed | -12,075 | 4,870 |

```
T-TEST GROUPS=historico_aborto(0 1)
  /MISSING=ANALYSIS
  /VARIABLES=estado_geral vitalidade asp_sociais saude_mental
  /CRITERIA=CI(.95).
```

# T-Test

**Notes**

| | | |
|---|---|---|
| Output Created | | 08-MAY-2020 11:43:14 |
| Comments | | |
| Input | Data | \\Mac\iCloud\Lavoro\FMABC\Orientações\Orientações\finite\Marina Rodrigues - Caio e Fabia\Marina Farias\data_article_6.sav |
| | Active Dataset | InsiemeDati1 |
| | Filter | <none> |
| | Weight | <none> |
| | Split File | <none> |
| | N of Rows in Working Data File | 106 |
| Missing Value Handling | Definition of Missing | User defined missing values are treated as missing. |
| | Cases Used | Statistics for each analysis are based on the cases with no missing or out-of-range data for any variable in the analysis. |
| Syntax | | T-TEST GROUPS=historico_aborto (0 1) /MISSING=ANALYSIS /VARIABLES=estado_geral vitalidade asp_sociais saude_mental /CRITERIA=CI(.95). |
| Resources | Processor Time | 00:00:00,02 |
| | Elapsed Time | 00:00:00,01 |

```
[InsiemeDati1] \\Mac\iCloud\Lavoro\FMABC\Orientações\Orientações\finite\Marin
a Rodrigues - Caio e Fabia\Marina Farias\data_article_6.sav
```

**Group Statistics**

| | historico_aborto | N | Mean | Std. Deviation | Std. Error Mean |
|---|---|---|---|---|---|
| estado_geral | nao | 86 | 60,406977 | 16,8043257 | 1,8120570 |
| | sim | 20 | 58,700000 | 19,5666203 | 4,3752293 |
| vitalidade | nao | 86 | 55,86 | 12,562 | 1,355 |
| | sim | 20 | 55,50 | 10,990 | 2,458 |
| asp_sociais | nao | 86 | 67,401 | 23,9816 | 2,5860 |
| | sim | 20 | 61,225 | 24,9660 | 5,5826 |
| saude_mental | nao | 86 | 59,06 | 19,333 | 2,085 |
| | sim | 20 | 60,35 | 18,534 | 4,144 |

**Independent Samples Test**

| | | Levene's Test for Equality of Variances | | t-test for Equality of Means | |
|---|---|---|---|---|---|
| | | F | Sig. | t | df |
| estado_geral | Equal variances assumed | ,211 | ,647 | ,397 | 104 |
| | Equal variances not assumed | | | ,360 | 25,907 |
| vitalidade | Equal variances assumed | 1,120 | ,292 | ,118 | 104 |
| | Equal variances not assumed | | | ,128 | 31,647 |
| asp_sociais | Equal variances assumed | ,000 | ,999 | 1,030 | 104 |
| | Equal variances not assumed | | | 1,004 | 27,743 |
| saude_mental | Equal variances assumed | ,069 | ,793 | -,271 | 104 |
| | Equal variances not assumed | | | -,278 | 29,411 |

**Independent Samples Test**

| | | t-test for Equality of Means | | |
|---|---|---|---|---|
| | | Sig. (2-tailed) | Mean Difference | Std. Error Difference |
| estado_geral | Equal variances assumed | ,693 | 1,7069767 | 4,3051102 |
| | Equal variances not assumed | ,721 | 1,7069767 | 4,7356290 |
| vitalidade | Equal variances assumed | ,906 | ,360 | 3,051 |
| | Equal variances not assumed | ,899 | ,360 | 2,806 |
| asp_sociais | Equal variances assumed | ,306 | 6,1762 | 5,9988 |
| | Equal variances not assumed | ,324 | 6,1762 | 6,1524 |
| saude_mental | Equal variances assumed | ,787 | -1,292 | 4,764 |
| | Equal variances not assumed | ,783 | -1,292 | 4,639 |

**Independent Samples Test**

| | | t-test for Equality of Means | |
|---|---|---|---|
| | | 95% Confidence Interval of the Difference | |
| | | Lower | Upper |
| estado_geral | Equal variances assumed | -6,8302184 | 10,2441719 |
| | Equal variances not assumed | -8,0289525 | 11,4429059 |
| vitalidade | Equal variances assumed | -5,690 | 6,411 |
| | Equal variances not assumed | -5,358 | 6,079 |
| asp_sociais | Equal variances assumed | -5,7197 | 18,0720 |
| | Equal variances not assumed | -6,4318 | 18,7841 |
| saude_mental | Equal variances assumed | -10,738 | 8,155 |
| | Equal variances not assumed | -10,774 | 8,190 |

```
T-TEST GROUPS=anticoncecpcional_prev(0 1)
 /MISSING=ANALYSIS
 /VARIABLES=estado_geral vitalidade asp_sociais saude_mental
 /CRITERIA=CI(.95).
```

# T-Test

**Notes**

| | | |
|---|---|---|
| Output Created | | 08-MAY-2020 11:43:45 |
| Comments | | |
| Input | Data | \\Mac\iCloud\Lavoro\FMABC\Orientações\Orientações\finite\Marina Rodrigues - Caio e Fabia\Marina Farias\data_article_6.sav |
| | Active Dataset | InsiemeDati1 |
| | Filter | <none> |
| | Weight | <none> |
| | Split File | <none> |
| | N of Rows in Working Data File | 106 |
| Missing Value Handling | Definition of Missing | User defined missing values are treated as missing. |
| | Cases Used | Statistics for each analysis are based on the cases with no missing or out-of-range data for any variable in the analysis. |
| Syntax | | T-TEST GROUPS=anticoncecpcional_prev(0 1)<br>/MISSING=ANALYSIS<br>/VARIABLES=estado_geral vitalidade asp_sociais saude_mental<br>/CRITERIA=CI(.95). |
| Resources | Processor Time | 00:00:00,00 |
| | Elapsed Time | 00:00:00,01 |

```
[InsiemeDati1] \\Mac\iCloud\Lavoro\FMABC\Orientações\Orientações\finite\Marin
a Rodrigues - Caio e Fabia\Marina Farias\data_article_6.sav
```

**Group Statistics**

| | anticoncecpcional_prev | N | Mean | Std. Deviation | Std. Error Mean |
|---|---|---|---|---|---|
| estado_geral | nao | 6 | 53,666667 | 5,1639778 | 2,1081851 |
| | sim | 100 | 60,470000 | 17,6752152 | 1,7675215 |
| vitalidade | nao | 6 | 54,17 | 10,685 | 4,362 |
| | sim | 100 | 55,89 | 12,360 | 1,236 |
| asp_sociais | nao | 6 | 60,417 | 12,2899 | 5,0173 |
| | sim | 100 | 66,585 | 24,6953 | 2,4695 |
| saude_mental | nao | 6 | 51,33 | 19,664 | 8,028 |
| | sim | 100 | 59,78 | 19,065 | 1,907 |

**Independent Samples Test**

| | | Levene's Test for Equality of Variances | | t-test for Equality of Means | |
|---|---|---|---|---|---|
| | | F | Sig. | t | df |
| estado_geral | Equal variances assumed | 5,019 | ,027 | -,937 | 104 |
| | Equal variances not assumed | | | -2,473 | 14,147 |
| vitalidade | Equal variances assumed | ,196 | ,659 | -,334 | 104 |
| | Equal variances not assumed | | | -,380 | 5,833 |
| asp_sociais | Equal variances assumed | 3,732 | ,056 | -,605 | 104 |
| | Equal variances not assumed | | | -1,103 | 7,693 |
| saude_mental | Equal variances assumed | ,000 | ,993 | -1,052 | 104 |
| | Equal variances not assumed | | | -1,024 | 5,579 |

**Independent Samples Test**

| | | t-test for Equality of Means | | |
|---|---|---|---|---|
| | | Sig. (2-tailed) | Mean Difference | Std. Error Difference |
| estado_geral | Equal variances assumed | ,351 | -6,8033333 | 7,2640203 |
| | Equal variances not assumed | ,027 | -6,8033333 | 2,7511046 |
| vitalidade | Equal variances assumed | ,739 | -1,723 | 5,163 |
| | Equal variances not assumed | ,717 | -1,723 | 4,534 |
| asp_sociais | Equal variances assumed | ,546 | -6,1683 | 10,1904 |
| | Equal variances not assumed | ,303 | -6,1683 | 5,5922 |
| saude_mental | Equal variances assumed | ,295 | -8,447 | 8,026 |
| | Equal variances not assumed | ,348 | -8,447 | 8,251 |

**Independent Samples Test**

| | | t-test for Equality of Means | |
|---|---|---|---|
| | | 95% Confidence Interval of the Difference | |
| | | Lower | Upper |
| estado_geral | Equal variances assumed | -21,2081581 | 7,6014914 |
| | Equal variances not assumed | -12,6981263 | -,9085404 |
| vitalidade | Equal variances assumed | -11,963 | 8,516 |
| | Equal variances not assumed | -12,895 | 9,448 |
| asp_sociais | Equal variances assumed | -26,3763 | 14,0397 |
| | Equal variances not assumed | -19,1539 | 6,8172 |
| saude_mental | Equal variances assumed | -24,362 | 7,469 |
| | Equal variances not assumed | -29,011 | 12,118 |

```
ONEWAY estado_geral vitalidade asp_sociais saude_mental BY dismenorreia
  /MISSING ANALYSIS.
```

# Oneway

**Notes**

| | | |
|---|---|---|
| Output Created | | 08-MAY-2020 11:51:23 |
| Comments | | |
| Input | Data | \\Mac\iCloud\Lavoro\FMABC\Orientações\Orientações\finite\Marina Rodrigues - Caio e Fabia\Marina Farias\data_article_6.sav |
| | Active Dataset | InsiemeDati1 |
| | Filter | <none> |
| | Weight | <none> |
| | Split File | <none> |
| | N of Rows in Working Data File | 106 |
| Missing Value Handling | Definition of Missing | User-defined missing values are treated as missing. |
| | Cases Used | Statistics for each analysis are based on cases with no missing data for any variable in the analysis. |
| Syntax | | ONEWAY estado_geral vitalidade asp_sociais saude_mental BY dismenorreia /MISSING ANALYSIS. |
| Resources | Processor Time | 00:00:00,02 |
| | Elapsed Time | 00:00:00,02 |

```
[InsiemeDati1] \\Mac\iCloud\Lavoro\FMABC\Orientações\Orientações\finite\Marin
a Rodrigues - Caio e Fabia\Marina Farias\data_article_6.sav
```

**ANOVA**

| | | Sum of Squares | df | Mean Square | F | Sig. |
|---|---|---|---|---|---|---|
| estado_geral | Between Groups | 1506,044 | 2 | 753,022 | 2,601 | ,079 |
| | Within Groups | 29818,192 | 103 | 289,497 | | |
| | Total | 31324,236 | 105 | | | |
| vitalidade | Between Groups | 131,177 | 2 | 65,589 | ,434 | ,649 |
| | Within Groups | 15580,256 | 103 | 151,265 | | |
| | Total | 15711,434 | 105 | | | |
| asp_sociais | Between Groups | 2054,925 | 2 | 1027,463 | 1,785 | ,173 |
| | Within Groups | 59291,679 | 103 | 575,647 | | |
| | Total | 61346,604 | 105 | | | |
| saude_mental | Between Groups | 35,301 | 2 | 17,651 | ,047 | ,954 |
| | Within Groups | 38287,038 | 103 | 371,719 | | |
| | Total | 38322,340 | 105 | | | |

```
ONEWAY estado_geral vitalidade asp_sociais saude_mental BY grau_dor
  /MISSING ANALYSIS.
```

# Oneway

**Notes**

| | | |
|---|---|---|
| Output Created | | 08-MAY-2020 11:52:01 |
| Comments | | |
| Input | Data | \\Mac\iCloud\Lavoro\FMABC\Orientações\Orientações\finite\Marina Rodrigues - Caio e Fabia\Marina Farias\data_article_6.sav |
| | Active Dataset | InsiemeDati1 |
| | Filter | <none> |
| | Weight | <none> |
| | Split File | <none> |
| | N of Rows in Working Data File | 106 |
| Missing Value Handling | Definition of Missing | User-defined missing values are treated as missing. |
| | Cases Used | Statistics for each analysis are based on cases with no missing data for any variable in the analysis. |
| Syntax | | ONEWAY estado_geral vitalidade asp_sociais saude_mental BY grau_dor /MISSING ANALYSIS. |
| Resources | Processor Time | 00:00:00,00 |
| | Elapsed Time | 00:00:00,01 |

```
[InsiemeDati1] \\Mac\iCloud\Lavoro\FMABC\Orientações\Orientações\finite\Marina Rodrigues - Caio e Fabia\Marina Farias\data_article_6.sav
```

**ANOVA**

| | | Sum of Squares | df | Mean Square | F | Sig. |
|---|---|---|---|---|---|---|
| estado_geral | Between Groups | 2334,455 | 4 | 583,614 | 2,033 | ,095 |
| | Within Groups | 28989,780 | 101 | 287,028 | | |
| | Total | 31324,236 | 105 | | | |
| vitalidade | Between Groups | 382,570 | 4 | 95,642 | ,630 | ,642 |
| | Within Groups | 15328,864 | 101 | 151,771 | | |
| | Total | 15711,434 | 105 | | | |
| asp_sociais | Between Groups | 4113,182 | 4 | 1028,295 | 1,815 | ,132 |
| | Within Groups | 57233,422 | 101 | 566,668 | | |
| | Total | 61346,604 | 105 | | | |
| saude_mental | Between Groups | 864,246 | 4 | 216,061 | ,583 | ,676 |
| | Within Groups | 37458,094 | 101 | 370,872 | | |
| | Total | 38322,340 | 105 | | | |

```
ONEWAY estado_geral vitalidade asp_sociais saude_mental BY dispaurenia
  /MISSING ANALYSIS.
```

# Oneway

**Notes**

| | | |
|---|---|---|
| Output Created | | 08-MAY-2020 11:52:52 |
| Comments | | |
| Input | Data | \\Mac\iCloud\Lavoro\FMABC\Orientações\Orientações\finite\Marina Rodrigues - Caio e Fabia\Marina Farias\data_article_6.sav |
| | Active Dataset | InsiemeDati1 |
| | Filter | <none> |
| | Weight | <none> |
| | Split File | <none> |
| | N of Rows in Working Data File | 106 |
| Missing Value Handling | Definition of Missing | User-defined missing values are treated as missing. |
| | Cases Used | Statistics for each analysis are based on cases with no missing data for any variable in the analysis. |
| Syntax | | ONEWAY estado_geral vitalidade asp_sociais saude_mental BY dispaurenia<br>/MISSING ANALYSIS. |
| Resources | Processor Time | 00:00:00,02 |
| | Elapsed Time | 00:00:00,01 |

```
[InsiemeDati1] \\Mac\iCloud\Lavoro\FMABC\Orientações\Orientações\finite\Marina Rodrigues - Caio e Fabia\Marina Farias\data_article_6.sav
```

**ANOVA**

| | | Sum of Squares | df | Mean Square | F | Sig. |
|---|---|---|---|---|---|---|
| estado_geral | Between Groups | 4676,076 | 3 | 1558,692 | 5,966 | ,001 |
| | Within Groups | 26648,160 | 102 | 261,256 | | |
| | Total | 31324,236 | 105 | | | |
| vitalidade | Between Groups | 696,743 | 3 | 232,248 | 1,578 | ,199 |
| | Within Groups | 15014,690 | 102 | 147,203 | | |
| | Total | 15711,434 | 105 | | | |
| asp_sociais | Between Groups | 849,613 | 3 | 283,204 | ,477 | ,699 |
| | Within Groups | 60496,991 | 102 | 593,108 | | |
| | Total | 61346,604 | 105 | | | |
| saude_mental | Between Groups | 1908,347 | 3 | 636,116 | 1,782 | ,155 |
| | Within Groups | 36413,993 | 102 | 357,000 | | |
| | Total | 38322,340 | 105 | | | |

```
SORT CASES  BY dispaurenia.
```

```
SPLIT FILE LAYERED BY dispaurenia.
FREQUENCIES VARIABLES=estado_geral
  /FORMAT=NOTABLE
  /STATISTICS=STDDEV MEAN
  /ORDER=ANALYSIS.
```

# Frequencies

**Notes**

| | | |
|---|---|---|
| Output Created | | 08-MAY-2020 11:56:58 |
| Comments | | |
| Input | Data | \\Mac\iCloud\Lavoro\FMABC\Orientações\Orientações\finite\Marina Rodrigues - Caio e Fabia\Marina Farias\data_article_6.sav |
| | Active Dataset | InsiemeDati1 |
| | Filter | <none> |
| | Weight | <none> |
| | Split File | dispaurenia |
| | N of Rows in Working Data File | 106 |
| Missing Value Handling | Definition of Missing | User-defined missing values are treated as missing. |
| | Cases Used | Statistics are based on all cases with valid data. |
| Syntax | | FREQUENCIES VARIABLES=estado_geral<br>/FORMAT=NOTABLE<br>/STATISTICS=STDDEV MEAN<br>/ORDER=ANALYSIS. |
| Resources | Processor Time | 00:00:00,02 |
| | Elapsed Time | 00:00:00,00 |

```
[InsiemeDati1] \\Mac\iCloud\Lavoro\FMABC\Orientações\Orientações\finite\Marina Rodrigues - Caio e Fabia\Marina Farias\data_article_6.sav
```

**Statistics**

estado_geral

| | | | |
|---|---|---|---|
| Ausente | N | Valid | 56 |
| | | Missing | 0 |
| | Mean | | 65,375000 |
| | Std. Deviation | | 16,1566901 |
| Penetração | N | Valid | 13 |
| | | Missing | 0 |
| | Mean | | 48,384615 |
| | Std. Deviation | | 13,1626901 |
| Profundidade | N | Valid | 24 |
| | | Missing | 0 |
| | Mean | | 59,541667 |
| | Std. Deviation | | 16,5266035 |
| Penetraçao e Profundiddade | N | Valid | 13 |
| | | Missing | 0 |
| | Mean | | 50,000000 |
| | Std. Deviation | | 18,0969611 |

```
 SPLIT FILE OFF.
T-TEST GROUPS=dor_fora_menst(0 1)
  /MISSING=ANALYSIS
  /VARIABLES=estado_geral vitalidade asp_sociais saude_mental
  /CRITERIA=CI(.95).
```

# T-Test

**Notes**

| | | |
|---|---|---|
| Output Created | | 08-MAY-2020 11:59:59 |
| Comments | | |
| Input | Data | \\Mac\iCloud\Lavoro\FMABC\Orientações\Orientações\finite\Marina Rodrigues - Caio e Fabia\Marina Farias\data_article_6.sav |
| | Active Dataset | InsiemeDati1 |
| | Filter | <none> |
| | Weight | <none> |
| | Split File | <none> |
| | N of Rows in Working Data File | 106 |
| Missing Value Handling | Definition of Missing | User defined missing values are treated as missing. |
| | Cases Used | Statistics for each analysis are based on the cases with no missing or out-of-range data for any variable in the analysis. |
| Syntax | | T-TEST GROUPS=dor_fora_menst (0 1) /MISSING=ANALYSIS /VARIABLES=estado_geral vitalidade asp_sociais saude_mental /CRITERIA=CI(.95). |
| Resources | Processor Time | 00:00:00,00 |
| | Elapsed Time | 00:00:00,01 |

```
[InsiemeDati1] \\Mac\iCloud\Lavoro\FMABC\Orientações\Orientações\finite\Marin
a Rodrigues - Caio e Fabia\Marina Farias\data_article_6.sav
```

**Group Statistics**

| | dor_fora_menst | N | Mean | Std. Deviation | Std. Error Mean |
|---|---|---|---|---|---|
| estado_geral | não | 85 | 60,423529 | 17,1077652 | 1,8555977 |
| | sim | 21 | 58,714286 | 18,2897317 | 3,9911467 |
| vitalidade | não | 85 | 55,76 | 12,163 | 1,319 |
| | sim | 21 | 55,90 | 12,814 | 2,796 |
| asp_sociais | não | 85 | 66,135 | 23,7755 | 2,5788 |
| | sim | 21 | 66,643 | 26,3240 | 5,7444 |
| saude_mental | não | 85 | 59,46 | 19,162 | 2,078 |
| | sim | 21 | 58,67 | 19,324 | 4,217 |

**Independent Samples Test**

| | | Levene's Test for Equality of Variances | | t-test for Equality of Means | |
|---|---|---|---|---|---|
| | | F | Sig. | t | df |
| estado_geral | Equal variances assumed | ,022 | ,881 | ,404 | 104 |
| | Equal variances not assumed | | | ,388 | 29,255 |
| vitalidade | Equal variances assumed | ,144 | ,706 | -,047 | 104 |
| | Equal variances not assumed | | | -,045 | 29,547 |
| asp_sociais | Equal variances assumed | ,089 | ,766 | -,086 | 104 |
| | Equal variances not assumed | | | -,081 | 28,597 |
| saude_mental | Equal variances assumed | ,011 | ,916 | ,169 | 104 |
| | Equal variances not assumed | | | ,168 | 30,469 |

**Independent Samples Test**

| | | t-test for Equality of Means | | |
|---|---|---|---|---|
| | | Sig. (2-tailed) | Mean Difference | Std. Error Difference |
| estado_geral | Equal variances assumed | ,687 | 1,7092437 | 4,2258695 |
| | Equal variances not assumed | ,701 | 1,7092437 | 4,4014196 |
| vitalidade | Equal variances assumed | ,963 | -,140 | 2,995 |
| | Equal variances not assumed | ,964 | -,140 | 3,092 |
| asp_sociais | Equal variances assumed | ,932 | -,5076 | 5,9183 |
| | Equal variances not assumed | ,936 | -,5076 | 6,2967 |
| saude_mental | Equal variances assumed | ,866 | ,792 | 4,677 |
| | Equal variances not assumed | ,867 | ,792 | 4,701 |

**Independent Samples Test**

| | | t-test for Equality of Means | |
|---|---|---|---|
| | | 95% Confidence Interval of the Difference | |
| | | Lower | Upper |
| estado_geral | Equal variances assumed | -6,6708141 | 10,0893015 |
| | Equal variances not assumed | -7,2892582 | 10,7077456 |
| vitalidade | Equal variances assumed | -6,080 | 5,799 |
| | Equal variances not assumed | -6,458 | 6,178 |
| asp_sociais | Equal variances assumed | -12,2438 | 11,2287 |
| | Equal variances not assumed | -13,3936 | 12,3785 |
| saude_mental | Equal variances assumed | -8,483 | 10,067 |
| | Equal variances not assumed | -8,803 | 10,387 |

```
T-TEST GROUPS=alt_intestinais(0 1)
  /MISSING=ANALYSIS
  /VARIABLES=estado_geral vitalidade asp_sociais saude_mental
  /CRITERIA=CI(.95).
```

# T-Test

**Notes**

| | | |
|---|---|---|
| Output Created | | 08-MAY-2020 12:00:25 |
| Comments | | |
| Input | Data | \\Mac\iCloud\Lavoro\FMABC\Orientações\Orientações\finite\Marina Rodrigues - Caio e Fabia\Marina Farias\data_article_6.sav |
| | Active Dataset | InsiemeDati1 |
| | Filter | <none> |
| | Weight | <none> |
| | Split File | <none> |
| | N of Rows in Working Data File | 106 |
| Missing Value Handling | Definition of Missing | User defined missing values are treated as missing. |
| | Cases Used | Statistics for each analysis are based on the cases with no missing or out-of-range data for any variable in the analysis. |
| Syntax | | T-TEST GROUPS=alt_intestinais(0 1)<br>/MISSING=ANALYSIS<br>/VARIABLES=estado_geral vitalidade asp_sociais saude_mental<br>/CRITERIA=CI(.95). |
| Resources | Processor Time | 00:00:00,02 |
| | Elapsed Time | 00:00:00,02 |

```
[InsiemeDati1] \\Mac\iCloud\Lavoro\FMABC\Orientações\Orientações\finite\Marin
a Rodrigues - Caio e Fabia\Marina Farias\data_article_6.sav
```

**Group Statistics**

| | alt_intestinais | N | Mean | Std. Deviation | Std. Error Mean |
|---|---|---|---|---|---|
| estado_geral | não | 29 | 58,310345 | 21,0477407 | 3,9084673 |
| | sim | 77 | 60,753247 | 15,7255707 | 1,7920951 |
| vitalidade | não | 29 | 56,90 | 13,257 | 2,462 |
| | sim | 77 | 55,38 | 11,889 | 1,355 |
| asp_sociais | não | 29 | 67,672 | 24,2101 | 4,4957 |
| | sim | 77 | 65,695 | 24,2933 | 2,7685 |
| saude_mental | não | 29 | 60,00 | 20,840 | 3,870 |
| | sim | 77 | 59,04 | 18,547 | 2,114 |

**Independent Samples Test**

| | | Levene's Test for Equality of Variances | | t-test for Equality of Means | |
|---|---|---|---|---|---|
| | | F | Sig. | t | df |
| estado_geral | Equal variances assumed | 3,536 | ,063 | -,647 | 104 |
| | Equal variances not assumed | | | -,568 | 40,354 |
| vitalidade | Equal variances assumed | ,962 | ,329 | ,568 | 104 |
| | Equal variances not assumed | | | ,541 | 45,978 |
| asp_sociais | Equal variances assumed | ,080 | ,778 | ,374 | 104 |
| | Equal variances not assumed | | | ,375 | 50,583 |
| saude_mental | Equal variances assumed | ,840 | ,361 | ,230 | 104 |
| | Equal variances not assumed | | | ,218 | 45,699 |

**Independent Samples Test**

| | | t-test for Equality of Means | | |
|---|---|---|---|---|
| | | Sig. (2-tailed) | Mean Difference | Std. Error Difference |
| estado_geral | Equal variances assumed | ,519 | -2,4429019 | 3,7736263 |
| | Equal variances not assumed | ,573 | -2,4429019 | 4,2997351 |
| vitalidade | Equal variances assumed | ,571 | 1,520 | 2,674 |
| | Equal variances not assumed | ,591 | 1,520 | 2,810 |
| asp_sociais | Equal variances assumed | ,709 | 1,9776 | 5,2880 |
| | Equal variances not assumed | ,710 | 1,9776 | 5,2798 |
| saude_mental | Equal variances assumed | ,819 | ,961 | 4,181 |
| | Equal variances not assumed | ,828 | ,961 | 4,409 |

**Independent Samples Test**

| | | t-test for Equality of Means<br>95% Confidence Interval of the Difference | |
|---|---|---|---|
| | | Lower | Upper |
| estado_geral | Equal variances assumed | -9,9261445 | 5,0403406 |
| | Equal variances not assumed | -11,1306184 | 6,2448145 |
| vitalidade | Equal variances assumed | -3,782 | 6,822 |
| | Equal variances not assumed | -4,136 | 7,176 |
| asp_sociais | Equal variances assumed | -8,5088 | 12,4640 |
| | Equal variances not assumed | -8,6241 | 12,5793 |
| saude_mental | Equal variances assumed | -7,331 | 9,253 |
| | Equal variances not assumed | -7,916 | 9,838 |

```
T-TEST GROUPS=alt_urinaria(0 1)
 /MISSING=ANALYSIS
 /VARIABLES=estado_geral vitalidade asp_sociais saude_mental
 /CRITERIA=CI(.95).
```

# T-Test

**Notes**

| | | |
|---|---|---|
| Output Created | | 08-MAY-2020 12:00:50 |
| Comments | | |
| Input | Data | \\Mac\iCloud\Lavoro\FMABC\Orientações\Orientações\finite\Marina Rodrigues - Caio e Fabia\Marina Farias\data_article_6.sav |
| | Active Dataset | InsiemeDati1 |
| | Filter | <none> |
| | Weight | <none> |
| | Split File | <none> |
| | N of Rows in Working Data File | 106 |
| Missing Value Handling | Definition of Missing | User defined missing values are treated as missing. |
| | Cases Used | Statistics for each analysis are based on the cases with no missing or out-of-range data for any variable in the analysis. |
| Syntax | | T-TEST GROUPS=alt_urinaria(0 1) /MISSING=ANALYSIS /VARIABLES=estado_geral vitalidade asp_sociais saude_mental /CRITERIA=CI(.95). |
| Resources | Processor Time | 00:00:00,02 |
| | Elapsed Time | 00:00:00,01 |

```
[InsiemeDati1] \\Mac\iCloud\Lavoro\FMABC\Orientações\Orientações\finite\Marina Rodrigues - Caio e Fabia\Marina Farias\data_article_6.sav
```

**Group Statistics**

| | alt_urinaria | N | Mean | Std. Deviation | Std. Error Mean |
|---|---|---|---|---|---|
| estado_geral | não | 101 | 59,990099 | 17,3651922 | 1,7279012 |
| | sim | 5 | 62,000000 | 16,9558250 | 7,5828754 |
| vitalidade | não | 101 | 55,50 | 12,218 | 1,216 |
| | sim | 5 | 61,80 | 12,194 | 5,453 |
| asp_sociais | não | 101 | 66,168 | 24,4114 | 2,4290 |
| | sim | 5 | 67,600 | 20,8878 | 9,3413 |
| saude_mental | não | 101 | 59,11 | 18,802 | 1,871 |
| | sim | 5 | 63,20 | 26,892 | 12,027 |

**Independent Samples Test**

| | | Levene's Test for Equality of Variances | | t-test for Equality of Means | |
|---|---|---|---|---|---|
| | | F | Sig. | t | df |
| estado_geral | Equal variances assumed | ,109 | ,742 | -,253 | 104 |
| | Equal variances not assumed | | | -,258 | 4,426 |
| vitalidade | Equal variances assumed | ,005 | ,945 | -1,126 | 104 |
| | Equal variances not assumed | | | -1,128 | 4,407 |
| asp_sociais | Equal variances assumed | ,599 | ,441 | -,129 | 104 |
| | Equal variances not assumed | | | -,148 | 4,558 |
| saude_mental | Equal variances assumed | ,813 | ,369 | -,466 | 104 |
| | Equal variances not assumed | | | -,336 | 4,196 |

**Independent Samples Test**

| | | t-test for Equality of Means | | |
|---|---|---|---|---|
| | | Sig. (2-tailed) | Mean Difference | Std. Error Difference |
| estado_geral | Equal variances assumed | ,801 | -2,0099010 | 7,9487229 |
| | Equal variances not assumed | ,808 | -2,0099010 | 7,7772516 |
| vitalidade | Equal variances assumed | ,263 | -6,305 | 5,597 |
| | Equal variances not assumed | ,317 | -6,305 | 5,587 |
| asp_sociais | Equal variances assumed | ,898 | -1,4317 | 11,1263 |
| | Equal variances not assumed | ,888 | -1,4317 | 9,6520 |
| saude_mental | Equal variances assumed | ,642 | -4,091 | 8,785 |
| | Equal variances not assumed | ,753 | -4,091 | 12,171 |

**Independent Samples Test**

| | | t-test for Equality of Means | |
|---|---|---|---|
| | | 95% Confidence Interval of the Difference | |
| | | Lower | Upper |
| estado_geral | Equal variances assumed | -17,7725169 | 13,7527149 |
| | Equal variances not assumed | -22,8080390 | 18,7882370 |
| vitalidade | Equal variances assumed | -17,404 | 4,794 |
| | Equal variances not assumed | -21,269 | 8,659 |
| asp_sociais | Equal variances assumed | -23,4956 | 20,6322 |
| | Equal variances not assumed | -26,9835 | 24,1202 |
| saude_mental | Equal variances assumed | -21,513 | 13,331 |
| | Equal variances not assumed | -37,271 | 29,089 |

```
 *Nonparametric Tests: Independent Samples.
NPTESTS
  /INDEPENDENT TEST (cap_func lim_fisico dor lim_emocional) GROUP (tipo_infert) MANN_WHITNEY
  /MISSING SCOPE=ANALYSIS USERMISSING=EXCLUDE
  /CRITERIA ALPHA=0.05  CILEVEL=95.
```

# Nonparametric Tests

**Notes**

| | | |
|---|---|---|
| Output Created | | 08-MAY-2020 12:02:58 |
| Comments | | |
| Input | Data | \\Mac\iCloud\Lavoro\FMABC\Orientações\Orientações\finite\Marina Rodrigues - Caio e Fabia\Marina Farias\data_article_6.sav |
| | Active Dataset | InsiemeDati1 |
| | Filter | <none> |
| | Weight | <none> |
| | Split File | <none> |
| | N of Rows in Working Data File | 106 |
| Syntax | | NPTESTS /INDEPENDENT TEST (cap_func lim_fisico dor lim_emocional) GROUP (tipo_infert) MANN_WHITNEY /MISSING SCOPE=ANALYSIS USERMISSING=EXCLUDE /CRITERIA ALPHA=0.05 CILEVEL=95. |
| Resources | Processor Time | 00:00:00,06 |
| | Elapsed Time | 00:00:00,09 |

```
[InsiemeDati1] \\Mac\iCloud\Lavoro\FMABC\Orientações\Orientações\finite\Marin
a Rodrigues - Caio e Fabia\Marina Farias\data_article_6.sav
```

## Hypothesis Test Summary

| | Null Hypothesis | Test | Sig. | Decision |
|---|---|---|---|---|
| 1 | The distribution of SF-36 - capacidade funcional is the same across categories of tipo inifertilidade. | Independent -Samples Mann-Whitney U Test | ,313 | Retain the null hypothesis. |
| 2 | The distribution of SF-36 - limitacao fisica is the same across categories of tipo inifertilidade. | Independent -Samples Mann-Whitney U Test | ,494 | Retain the null hypothesis. |
| 3 | The distribution of SF-36 - dor is the same across categories of tipo inifertilidade. | Independent -Samples Mann-Whitney U Test | ,244 | Retain the null hypothesis. |
| 4 | The distribution of SF-36 - limites emocionais is the same across categories of tipo inifertilidade. | Independent -Samples Mann-Whitney U Test | ,276 | Retain the null hypothesis. |

Asymptotic significances are displayed. The significance level is ,05.

```
 *Nonparametric Tests: Independent Samples.
NPTESTS
  /INDEPENDENT TEST (cap_func lim_fisico dor lim_emocional) GROUP (historico_aborto) MANN_WHITN
  /MISSING SCOPE=ANALYSIS USERMISSING=EXCLUDE
  /CRITERIA ALPHA=0.05  CILEVEL=95.
```

# Nonparametric Tests

**Notes**

| | | |
|---|---|---|
| Output Created | | 08-MAY-2020 12:03:17 |
| Comments | | |
| Input | Data | \\Mac\iCloud\Lavoro\FMABC\Orientações\Orientações\finite\Marina Rodrigues - Caio e Fabia\Marina Farias\data_article_6.sav |
| | Active Dataset | InsiemeDati1 |
| | Filter | <none> |
| | Weight | <none> |
| | Split File | <none> |
| | N of Rows in Working Data File | 106 |
| Syntax | | NPTESTS<br>/INDEPENDENT TEST (cap_func lim_fisico dor lim_emocional) GROUP (historico_aborto) MANN_WHITNEY<br>/MISSING SCOPE=ANALYSIS USERMISSING=EXCLUDE<br>/CRITERIA ALPHA=0.05 CILEVEL=95. |
| Resources | Processor Time | 00:00:00,05 |
| | Elapsed Time | 00:00:00,09 |

```
[InsiemeDati1] \\Mac\iCloud\Lavoro\FMABC\Orientações\Orientações\finite\Marin
a Rodrigues - Caio e Fabia\Marina Farias\data_article_6.sav
```

**Hypothesis Test Summary**

| | Null Hypothesis | Test | Sig. | Decision |
|---|---|---|---|---|
| **1** | The distribution of SF-36 - capacidade funcional is the same across categories of historico aborto. | Independent -Samples Mann-Whitney U Test | ,778 | Retain the null hypothesis. |
| **2** | The distribution of SF-36 - limitacao fisica is the same across categories of historico aborto. | Independent -Samples Mann-Whitney U Test | ,272 | Retain the null hypothesis. |
| **3** | The distribution of SF-36 - dor is the same across categories of historico aborto. | Independent -Samples Mann-Whitney U Test | ,356 | Retain the null hypothesis. |
| **4** | The distribution of SF-36 - limites emocionais is the same across categories of historico aborto. | Independent -Samples Mann-Whitney U Test | ,935 | Retain the null hypothesis. |

Asymptotic significances are displayed. The significance level is ,05.

```
 *Nonparametric Tests: Independent Samples.
NPTESTS
  /INDEPENDENT TEST (cap_func lim_fisico dor lim_emocional) GROUP (anticoncecpcional_prev) MANN
  /MISSING SCOPE=ANALYSIS USERMISSING=EXCLUDE
  /CRITERIA ALPHA=0.05  CILEVEL=95.
```

## Nonparametric Tests

**Notes**

| | | |
|---|---|---|
| Output Created | | 08-MAY-2020 12:03:31 |
| Comments | | |
| Input | Data | \\Mac\iCloud\Lavoro\FMABC\Orientações\Orientações\finite\Marina Rodrigues - Caio e Fabia\Marina Farias\data_article_6.sav |
| | Active Dataset | InsiemeDati1 |
| | Filter | <none> |
| | Weight | <none> |
| | Split File | <none> |
| | N of Rows in Working Data File | 106 |
| Syntax | | NPTESTS<br>/INDEPENDENT TEST (cap_func lim_fisico dor lim_emocional) GROUP (anticoncecpcional_prev) MANN_WHITNEY<br>/MISSING SCOPE=ANALYSIS USERMISSING=EXCLUDE<br>/CRITERIA ALPHA=0.05 CILEVEL=95. |
| Resources | Processor Time | 00:00:00,03 |
| | Elapsed Time | 00:00:00,10 |

```
[InsiemeDati1] \\Mac\iCloud\Lavoro\FMABC\Orientações\Orientações\finite\Marin
a Rodrigues - Caio e Fabia\Marina Farias\data_article_6.sav
```

**Hypothesis Test Summary**

| | Null Hypothesis | Test | Sig. | Decision |
|---|---|---|---|---|
| **1** | The distribution of SF-36 - capacidade funcional is the same across categories of uso prévio AC. | Independent-Samples Mann-Whitney U Test | ,340 | Retain the null hypothesis. |
| **2** | The distribution of SF-36 - limitacao fisica is the same across categories of uso prévio AC. | Independent-Samples Mann-Whitney U Test | ,063 | Retain the null hypothesis. |
| **3** | The distribution of SF-36 - dor is the same across categories of uso prévio AC. | Independent-Samples Mann-Whitney U Test | ,057 | Retain the null hypothesis. |
| **4** | The distribution of SF-36 - limites emocionais is the same across categories of uso prévio AC. | Independent-Samples Mann-Whitney U Test | ,020 | Reject the null hypothesis. |

Asymptotic significances are displayed. The significance level is ,05.

```
 SORT CASES  BY anticoncecpcional_prev.
SPLIT FILE LAYERED BY anticoncecpcional_prev.
EXAMINE VARIABLES=lim_emocional
  /PLOT NONE
  /STATISTICS DESCRIPTIVES
  /CINTERVAL 95
  /MISSING LISTWISE
  /NOTOTAL.
```

# Explore

**Notes**

| | | |
|---|---|---|
| Output Created | | 08-MAY-2020 12:06:30 |
| Comments | | |
| Input | Data | \\Mac\iCloud\Lavoro\FMABC\Orientações\Orientações\finite\Marina Rodrigues - Caio e Fabia\Marina Farias\data_article_6.sav |
| | Active Dataset | InsiemeDati1 |
| | Filter | <none> |
| | Weight | <none> |
| | Split File | anticoncecpcional_prev |
| | N of Rows in Working Data File | 106 |
| Missing Value Handling | Definition of Missing | User-defined missing values for dependent variables are treated as missing. |
| | Cases Used | Statistics are based on cases with no missing values for any dependent variable or factor used. |
| Syntax | | EXAMINE VARIABLES=lim_emocional<br>/PLOT NONE<br>/STATISTICS DESCRIPTIVES<br>/CINTERVAL 95<br>/MISSING LISTWISE<br>/NOTOTAL. |
| Resources | Processor Time | 00:00:00,00 |
| | Elapsed Time | 00:00:00,00 |

```
[InsiemeDati1] \\Mac\iCloud\Lavoro\FMABC\Orientações\Orientações\finite\Marin
a Rodrigues - Caio e Fabia\Marina Farias\data_article_6.sav
```

**Case Processing Summary**

| anticoncecpcional_prev | | Cases | | | | | |
|---|---|---|---|---|---|---|---|
| | | Valid | | Missing | | Total | |
| | | N | Percent | N | Percent | N | Percent |
| nao | lim_emocional | 6 | 100,0% | 0 | 0,0% | 6 | 100,0% |
| sim | lim_emocional | 100 | 100,0% | 0 | 0,0% | 100 | 100,0% |

**Descriptives**

| anticoncecpcional_prev | | | | Statistic | Std. Error |
|---|---|---|---|---|---|
| nao | lim_emocional | Mean | | 22,222222 | 16,4804411 |
| | | 95% Confidence Interval for Mean | Lower Bound | -20,142100 | |
| | | | Upper Bound | 64,586545 | |
| | | 5% Trimmed Mean | | 19,135802 | |
| | | Median | | ,000000 | |
| | | Variance | | 1629,630 | |
| | | Std. Deviation | | 40,3686714 | |
| | | Minimum | | ,0000 | |
| | | Maximum | | 100,0000 | |
| | | Range | | 100,0000 | |
| | | Interquartile Range | | 50,0000 | |
| | | Skewness | | 1,952 | ,845 |
| | | Kurtosis | | 3,657 | 1,741 |
| sim | lim_emocional | Mean | | 65,303333 | 3,9616090 |
| | | 95% Confidence Interval for Mean | Lower Bound | 57,442642 | |
| | | | Upper Bound | 73,164025 | |
| | | 5% Trimmed Mean | | 67,003704 | |
| | | Median | | 67,000000 | |
| | | Variance | | 1569,435 | |
| | | Std. Deviation | | 39,6160896 | |
| | | Minimum | | ,0000 | |
| | | Maximum | | 100,0000 | |
| | | Range | | 100,0000 | |
| | | Interquartile Range | | 66,6667 | |
| | | Skewness | | -,619 | ,241 |
| | | Kurtosis | | -1,210 | ,478 |

```
 SPLIT FILE OFF.
*Nonparametric Tests: Independent Samples.
NPTESTS
  /INDEPENDENT TEST (cap_func lim_fisico dor lim_emocional) GROUP (dismenorreia) KRUSKAL_WALLIS
  /MISSING SCOPE=ANALYSIS USERMISSING=EXCLUDE
  /CRITERIA ALPHA=0.05  CILEVEL=95.
```

# Nonparametric Tests

**Notes**

| | | |
|---|---|---|
| Output Created | | 08-MAY-2020 12:09:13 |
| Comments | | |
| Input | Data | \\Mac\iCloud\Lavoro\FMABC\Orientações\Orientações\finite\Marina Rodrigues - Caio e Fabia\Marina Farias\data_article_6.sav |
| | Active Dataset | InsiemeDati1 |
| | Filter | <none> |
| | Weight | <none> |
| | Split File | <none> |
| | N of Rows in Working Data File | 106 |
| Syntax | | NPTESTS /INDEPENDENT TEST (cap_func lim_fisico dor lim_emocional) GROUP (dismenorreia) KRUSKAL_WALLIS (COMPARE=PAIRWISE) /MISSING SCOPE=ANALYSIS USERMISSING=EXCLUDE /CRITERIA ALPHA=0.05 CILEVEL=95. |
| Resources | Processor Time | 00:00:00,08 |
| | Elapsed Time | 00:00:00,10 |

```
[InsiemeDati1] \\Mac\iCloud\Lavoro\FMABC\Orientações\Orientações\finite\Marin
a Rodrigues - Caio e Fabia\Marina Farias\data_article_6.sav
```

**Hypothesis Test Summary**

| | Null Hypothesis | Test | Sig. | Decision |
|---|---|---|---|---|
| 1 | The distribution of SF-36 - capacidade funcional is the same across categories of Dismenorreia. | Independent-Samples Kruskal-Wallis Test | ,063 | Retain the null hypothesis. |
| 2 | The distribution of SF-36 - limitacao fisica is the same across categories of Dismenorreia. | Independent-Samples Kruskal-Wallis Test | ,066 | Retain the null hypothesis. |
| 3 | The distribution of SF-36 - dor is the same across categories of Dismenorreia. | Independent-Samples Kruskal-Wallis Test | ,233 | Retain the null hypothesis. |
| 4 | The distribution of SF-36 - limites emocionais is the same across categories of Dismenorreia. | Independent-Samples Kruskal-Wallis Test | ,575 | Retain the null hypothesis. |

Asymptotic significances are displayed. The significance level is ,05.

```
 *Nonparametric Tests: Independent Samples.
NPTESTS
  /INDEPENDENT TEST (cap_func lim_fisico dor lim_emocional) GROUP (grau_dor) KRUSKAL_WALLIS(COM
  /MISSING SCOPE=ANALYSIS USERMISSING=EXCLUDE
  /CRITERIA ALPHA=0.05  CILEVEL=95.
```

## Nonparametric Tests

**Notes**

| | | |
|---|---|---|
| Output Created | | 08-MAY-2020 12:09:31 |
| Comments | | |
| Input | Data | \\Mac\iCloud\Lavoro\FMABC\Orientações\Orientações\finite\Marina Rodrigues - Caio e Fabia\Marina Farias\data_article_6.sav |
| | Active Dataset | InsiemeDati1 |
| | Filter | <none> |
| | Weight | <none> |
| | Split File | <none> |
| | N of Rows in Working Data File | 106 |
| Syntax | | NPTESTS /INDEPENDENT TEST (cap_func lim_fisico dor lim_emocional) GROUP (grau_dor) KRUSKAL_WALLIS (COMPARE=PAIRWISE) /MISSING SCOPE=ANALYSIS USERMISSING=EXCLUDE /CRITERIA ALPHA=0.05 CILEVEL=95. |
| Resources | Processor Time | 00:00:00,16 |
| | Elapsed Time | 00:00:00,18 |

```
[InsiemeDati1] \\Mac\iCloud\Lavoro\FMABC\Orientações\Orientações\finite\Marin
a Rodrigues - Caio e Fabia\Marina Farias\data_article_6.sav
```

**Hypothesis Test Summary**

| | Null Hypothesis | Test | Sig. | Decision |
|---|---|---|---|---|
| 1 | The distribution of SF-36 - capacidade funcional is the same across categories of Grau de Dor. | Independent-Samples Kruskal-Wallis Test | ,005 | Reject the null hypothesis. |
| 2 | The distribution of SF-36 - limitacao fisica is the same across categories of Grau de Dor. | Independent-Samples Kruskal-Wallis Test | ,011 | Reject the null hypothesis. |
| 3 | The distribution of SF-36 - dor is the same across categories of Grau de Dor. | Independent-Samples Kruskal-Wallis Test | ,054 | Retain the null hypothesis. |
| 4 | The distribution of SF-36 - limites emocionais is the same across categories of Grau de Dor. | Independent-Samples Kruskal-Wallis Test | ,076 | Retain the null hypothesis. |

Asymptotic significances are displayed. The significance level is ,05.

```
 SORT CASES  BY grau_dor.
SPLIT FILE LAYERED BY grau_dor.
EXAMINE VARIABLES=cap_func lim_fisico
  /PLOT NONE
  /STATISTICS DESCRIPTIVES
  /CINTERVAL 95
  /MISSING LISTWISE
  /NOTOTAL.
```

# Explore

**Notes**

| | | |
|---|---|---|
| Output Created | | 08-MAY-2020 12:16:00 |
| Comments | | |
| Input | Data | \\Mac\iCloud\Lavoro\FMABC\Orientações\Orientações\finite\Marina Rodrigues - Caio e Fabia\Marina Farias\data_article_6.sav |
| | Active Dataset | InsiemeDati1 |
| | Filter | <none> |
| | Weight | <none> |
| | Split File | grau_dor |
| | N of Rows in Working Data File | 106 |
| Missing Value Handling | Definition of Missing | User-defined missing values for dependent variables are treated as missing. |
| | Cases Used | Statistics are based on cases with no missing values for any dependent variable or factor used. |
| Syntax | | EXAMINE VARIABLES=cap_func lim_fisico<br>/PLOT NONE<br>/STATISTICS DESCRIPTIVES<br>/CINTERVAL 95<br>/MISSING LISTWISE<br>/NOTOTAL. |
| Resources | Processor Time | 00:00:00,02 |
| | Elapsed Time | 00:00:00,01 |

```
[InsiemeDati1] \\Mac\iCloud\Lavoro\FMABC\Orientações\Orientações\finite\Marin
a Rodrigues - Caio e Fabia\Marina Farias\data_article_6.sav
```

**Case Processing Summary**

| grau_dor | | Cases | | | | | |
|---|---|---|---|---|---|---|---|
| | | Valid | | Missing | | Total | |
| | | N | Percent | N | Percent | N | Percent |
| Ausente | cap_func | 17 | 100,0% | 0 | 0,0% | 17 | 100,0% |
| | lim_fisico | 17 | 100,0% | 0 | 0,0% | 17 | 100,0% |
| Leve | cap_func | 8 | 100,0% | 0 | 0,0% | 8 | 100,0% |
| | lim_fisico | 8 | 100,0% | 0 | 0,0% | 8 | 100,0% |
| Moderada | cap_func | 30 | 100,0% | 0 | 0,0% | 30 | 100,0% |
| | lim_fisico | 30 | 100,0% | 0 | 0,0% | 30 | 100,0% |
| Severa | cap_func | 41 | 100,0% | 0 | 0,0% | 41 | 100,0% |
| | lim_fisico | 41 | 100,0% | 0 | 0,0% | 41 | 100,0% |
| Incapacitante | cap_func | 10 | 100,0% | 0 | 0,0% | 10 | 100,0% |
| | lim_fisico | 10 | 100,0% | 0 | 0,0% | 10 | 100,0% |

**Descriptives**

| grau_dor | | | | Statistic | Std. Error |
|---|---|---|---|---|---|
| Ausente | cap_func | Mean | | 92,94 | 1,771 |
| | | 95% Confidence Interval for Mean | Lower Bound | 89,19 | |
| | | | Upper Bound | 96,70 | |
| | | 5% Trimmed Mean | | 93,55 | |
| | | Median | | 95,00 | |
| | | Variance | | 53,309 | |
| | | Std. Deviation | | 7,301 | |
| | | Minimum | | 75 | |
| | | Maximum | | 100 | |
| | | Range | | 25 | |
| | | Interquartile Range | | 10 | |
| | | Skewness | | -1,221 | ,550 |
| | | Kurtosis | | 1,098 | 1,063 |
| | lim_fisico | Mean | | 92,65 | 4,679 |
| | | 95% Confidence Interval for Mean | Lower Bound | 82,73 | |
| | | | Upper Bound | 102,57 | |
| | | 5% Trimmed Mean | | 96,00 | |
| | | Median | | 100,00 | |
| | | Variance | | 372,243 | |
| | | Std. Deviation | | 19,294 | |
| | | Minimum | | 25 | |
| | | Maximum | | 100 | |
| | | Range | | 75 | |
| | | Interquartile Range | | 0 | |
| | | Skewness | | -3,107 | ,550 |
| | | Kurtosis | | 10,261 | 1,063 |
| Leve | cap_func | Mean | | 61,88 | 7,004 |
| | | 95% Confidence Interval for Mean | Lower Bound | 45,31 | |
| | | | Upper Bound | 78,44 | |
| | | 5% Trimmed Mean | | 62,36 | |
| | | Median | | 62,50 | |
| | | Variance | | 392,411 | |
| | | Std. Deviation | | 19,809 | |
| | | Minimum | | 30 | |
| | | Maximum | | 85 | |
| | | Range | | 55 | |
| | | Interquartile Range | | 36 | |
| | | Skewness | | -,463 | ,752 |
| | | Kurtosis | | -,969 | 1,481 |

**Descriptives**

| grau_dor | | | | Statistic | Std. Error |
|---|---|---|---|---|---|
| | lim_fisico | Mean | | 43,75 | 16,870 |
| | | 95% Confidence Interval for Mean | Lower Bound | 3,86 | |
| | | | Upper Bound | 83,64 | |
| | | 5% Trimmed Mean | | 43,06 | |
| | | Median | | 37,50 | |
| | | Variance | | 2276,786 | |
| | | Std. Deviation | | 47,716 | |
| | | Minimum | | 0 | |
| | | Maximum | | 100 | |
| | | Range | | 100 | |
| | | Interquartile Range | | 94 | |
| | | Skewness | | ,144 | ,752 |
| | | Kurtosis | | -2,480 | 1,481 |
| Moderada | cap_func | Mean | | 83,50 | 3,516 |
| | | 95% Confidence Interval for Mean | Lower Bound | 76,31 | |
| | | | Upper Bound | 90,69 | |
| | | 5% Trimmed Mean | | 85,28 | |
| | | Median | | 87,50 | |
| | | Variance | | 370,948 | |
| | | Std. Deviation | | 19,260 | |
| | | Minimum | | 35 | |
| | | Maximum | | 100 | |
| | | Range | | 65 | |
| | | Interquartile Range | | 25 | |
| | | Skewness | | -1,229 | ,427 |
| | | Kurtosis | | ,858 | ,833 |
| | lim_fisico | Mean | | 79,17 | 5,882 |
| | | 95% Confidence Interval for Mean | Lower Bound | 67,14 | |
| | | | Upper Bound | 91,20 | |
| | | 5% Trimmed Mean | | 82,41 | |
| | | Median | | 100,00 | |
| | | Variance | | 1038,075 | |
| | | Std. Deviation | | 32,219 | |
| | | Minimum | | 0 | |
| | | Maximum | | 100 | |
| | | Range | | 100 | |
| | | Interquartile Range | | 50 | |
| | | Skewness | | -1,367 | ,427 |
| | | Kurtosis | | ,688 | ,833 |

**Descriptives**

| grau_dor | | | | Statistic | Std. Error |
|---|---|---|---|---|---|
| Severa | cap_func | Mean | | 79,73 | 3,147 |
| | | 95% Confidence Interval for Mean | Lower Bound | 73,37 | |
| | | | Upper Bound | 86,09 | |
| | | 5% Trimmed Mean | | 81,62 | |
| | | Median | | 85,00 | |
| | | Variance | | 405,951 | |
| | | Std. Deviation | | 20,148 | |
| | | Minimum | | 10 | |
| | | Maximum | | 100 | |
| | | Range | | 90 | |
| | | Interquartile Range | | 25 | |
| | | Skewness | | -1,344 | ,369 |
| | | Kurtosis | | 2,220 | ,724 |
| | lim_fisico | Mean | | 61,80 | 6,726 |
| | | 95% Confidence Interval for Mean | Lower Bound | 48,21 | |
| | | | Upper Bound | 75,40 | |
| | | 5% Trimmed Mean | | 63,12 | |
| | | Median | | 75,00 | |
| | | Variance | | 1854,811 | |
| | | Std. Deviation | | 43,068 | |
| | | Minimum | | 0 | |
| | | Maximum | | 100 | |
| | | Range | | 100 | |
| | | Interquartile Range | | 88 | |
| | | Skewness | | -,504 | ,369 |
| | | Kurtosis | | -1,584 | ,724 |
| Incapacitante | cap_func | Mean | | 84,20 | 4,079 |
| | | 95% Confidence Interval for Mean | Lower Bound | 74,97 | |
| | | | Upper Bound | 93,43 | |
| | | 5% Trimmed Mean | | 84,56 | |
| | | Median | | 85,00 | |
| | | Variance | | 166,400 | |
| | | Std. Deviation | | 12,900 | |
| | | Minimum | | 62 | |
| | | Maximum | | 100 | |
| | | Range | | 38 | |
| | | Interquartile Range | | 25 | |
| | | Skewness | | -,579 | ,687 |
| | | Kurtosis | | -,971 | 1,334 |

**Descriptives**

| grau_dor | | | | Statistic | Std. Error |
|---|---|---|---|---|---|
| lim_fisico | Mean | | | 79,50 | 11,510 |
| | 95% Confidence Interval for Mean | Lower Bound | | 53,46 | |
| | | Upper Bound | | 105,54 | |
| | 5% Trimmed Mean | | | 82,78 | |
| | Median | | | 100,00 | |
| | Variance | | | 1324,722 | |
| | Std. Deviation | | | 36,397 | |
| | Minimum | | | 0 | |
| | Maximum | | | 100 | |
| | Range | | | 100 | |
| | Interquartile Range | | | 34 | |
| | Skewness | | | -1,761 | ,687 |
| | Kurtosis | | | 1,859 | 1,334 |

```
 SPLIT FILE OFF.
*Nonparametric Tests: Independent Samples.
NPTESTS
  /INDEPENDENT TEST (cap_func lim_fisico dor lim_emocional) GROUP (dispaurenia) KRUSKAL_WALLIS(
  /MISSING SCOPE=ANALYSIS USERMISSING=EXCLUDE
  /CRITERIA ALPHA=0.05  CILEVEL=95.
```

## Nonparametric Tests

**Notes**

| | | |
|---|---|---|
| Output Created | | 08-MAY-2020 12:22:12 |
| Comments | | |
| Input | Data | \\Mac\iCloud\Lavoro\FMABC\Orientações\Orientações\finite\Marina Rodrigues - Caio e Fabia\Marina Farias\data_article_6.sav |
| | Active Dataset | InsiemeDati1 |
| | Filter | <none> |
| | Weight | <none> |
| | Split File | <none> |
| | N of Rows in Working Data File | 106 |
| Syntax | | NPTESTS /INDEPENDENT TEST (cap_func lim_fisico dor lim_emocional) GROUP (dispaurenia) KRUSKAL_WALLIS (COMPARE=PAIRWISE) /MISSING SCOPE=ANALYSIS USERMISSING=EXCLUDE /CRITERIA ALPHA=0.05 CILEVEL=95. |
| Resources | Processor Time | 00:00:00,06 |
| | Elapsed Time | 00:00:00,06 |

```
[InsiemeDati1] \\Mac\iCloud\Lavoro\FMABC\Orientações\Orientações\finite\Marina Rodrigues - Caio e Fabia\Marina Farias\data_article_6.sav
```

**Hypothesis Test Summary**

| | Null Hypothesis | Test | Sig. | Decision |
|---|---|---|---|---|
| 1 | The distribution of SF-36 - capacidade funcional is the same across categories of Dispareunia. | Independent-Samples Kruskal-Wallis Test | ,017 | Reject the null hypothesis. |
| 2 | The distribution of SF-36 - limitacao fisica is the same across categories of Dispareunia. | Independent-Samples Kruskal-Wallis Test | ,238 | Retain the null hypothesis. |
| 3 | The distribution of SF-36 - dor is the same across categories of Dispareunia. | Independent-Samples Kruskal-Wallis Test | ,102 | Retain the null hypothesis. |
| 4 | The distribution of SF-36 - limites emocionais is the same across categories of Dispareunia. | Independent-Samples Kruskal-Wallis Test | ,013 | Reject the null hypothesis. |

Asymptotic significances are displayed. The significance level is ,05.

```
 SORT CASES  BY dispaurenia.
SPLIT FILE LAYERED BY dispaurenia.
EXAMINE VARIABLES=cap_func lim_emocional
  /PLOT NONE
  /STATISTICS DESCRIPTIVES
  /CINTERVAL 95
  /MISSING LISTWISE
  /NOTOTAL.
```

# Explore

**Notes**

| | | |
|---|---|---|
| Output Created | | 08-MAY-2020 12:27:54 |
| Comments | | |
| Input | Data | \\Mac\iCloud\Lavoro\FMABC\Orientações\Orientações\finite\Marina Rodrigues - Caio e Fabia\Marina Farias\data_article_6.sav |
| | Active Dataset | InsiemeDati1 |
| | Filter | <none> |
| | Weight | <none> |
| | Split File | dispaurenia |
| | N of Rows in Working Data File | 106 |
| Missing Value Handling | Definition of Missing | User-defined missing values for dependent variables are treated as missing. |
| | Cases Used | Statistics are based on cases with no missing values for any dependent variable or factor used. |
| Syntax | | EXAMINE VARIABLES=cap_func lim_emocional<br>/PLOT NONE<br>/STATISTICS DESCRIPTIVES<br>/CINTERVAL 95<br>/MISSING LISTWISE<br>/NOTOTAL. |
| Resources | Processor Time | 00:00:00,00 |
| | Elapsed Time | 00:00:00,01 |

```
[InsiemeDati1] \\Mac\iCloud\Lavoro\FMABC\Orientações\Orientações\finite\Marin
a Rodrigues - Caio e Fabia\Marina Farias\data_article_6.sav
```

**Case Processing Summary**

| dispaurenia | | Cases | | | | |
|---|---|---|---|---|---|---|
| | | Valid | | Missing | | Total |
| | | N | Percent | N | Percent | N |
| Ausente | cap_func | 56 | 100,0% | 0 | 0,0% | 56 |
| | lim_emocional | 56 | 100,0% | 0 | 0,0% | 56 |
| Penetração | cap_func | 13 | 100,0% | 0 | 0,0% | 13 |
| | lim_emocional | 13 | 100,0% | 0 | 0,0% | 13 |
| Profundidade | cap_func | 24 | 100,0% | 0 | 0,0% | 24 |
| | lim_emocional | 24 | 100,0% | 0 | 0,0% | 24 |
| Penetraçao e Profundiddade | cap_func | 13 | 100,0% | 0 | 0,0% | 13 |
| | lim_emocional | 13 | 100,0% | 0 | 0,0% | 13 |

**Case Processing Summary**

| dispaurenia | | Cases Total Percent |
|---|---|---|
| Ausente | cap_func | 100,0% |
| | lim_emocional | 100,0% |
| Penetração | cap_func | 100,0% |
| | lim_emocional | 100,0% |
| Profundidade | cap_func | 100,0% |
| | lim_emocional | 100,0% |
| Penetraçao e Profundiddade | cap_func | 100,0% |
| | lim_emocional | 100,0% |

**Descriptives**

| dispaurenia | | | |
|---|---|---|---|
| Ausente | cap_func | Mean | |
| | | 95% Confidence Interval for Mean | Lower Bound |
| | | | Upper Bound |
| | | 5% Trimmed Mean | |
| | | Median | |
| | | Variance | |
| | | Std. Deviation | |
| | | Minimum | |
| | | Maximum | |
| | | Range | |
| | | Interquartile Range | |
| | | Skewness | |
| | | Kurtosis | |
| | lim_emocional | Mean | |
| | | 95% Confidence Interval for Mean | Lower Bound |
| | | | Upper Bound |
| | | 5% Trimmed Mean | |
| | | Median | |
| | | Variance | |
| | | Std. Deviation | |
| | | Minimum | |
| | | Maximum | |
| | | Range | |
| | | Interquartile Range | |
| | | Skewness | |
| | | Kurtosis | |
| Penetração | cap_func | Mean | |

**Descriptives**

| dispaurenia | | | | Statistic |
|---|---|---|---|---|
| Ausente | cap_func | Mean | | 85,71 |
| | | 95% Confidence Interval for Mean | Lower Bound | 81,02 |
| | | | Upper Bound | 90,40 |
| | | 5% Trimmed Mean | | 87,76 |
| | | Median | | 92,50 |
| | | Variance | | 306,753 |
| | | Std. Deviation | | 17,514 |
| | | Minimum | | 30 |
| | | Maximum | | 100 |
| | | Range | | 70 |
| | | Interquartile Range | | 20 |
| | | Skewness | | -1,608 |
| | | Kurtosis | | 2,215 |
| | lim_emocional | Mean | | 70,214286 |
| | | 95% Confidence Interval for Mean | Lower Bound | 60,785051 |
| | | | Upper Bound | 79,643520 |
| | | 5% Trimmed Mean | | 72,460317 |
| | | Median | | 83,333333 |
| | | Variance | | 1239,727 |
| | | Std. Deviation | | 35,2097569 |
| | | Minimum | | ,0000 |
| | | Maximum | | 100,0000 |
| | | Range | | 100,0000 |
| | | Interquartile Range | | 66,6667 |
| | | Skewness | | -,795 |
| | | Kurtosis | | -,712 |
| Penetração | cap_func | Mean | | 67,23 |

**Descriptives**

| dispaurenia | | | | Std. Error |
|---|---|---|---|---|
| Ausente | cap_func | Mean | | 2,340 |
| | | 95% Confidence Interval for Mean | Lower Bound | |
| | | | Upper Bound | |
| | | 5% Trimmed Mean | | |
| | | Median | | |
| | | Variance | | |
| | | Std. Deviation | | |
| | | Minimum | | |
| | | Maximum | | |
| | | Range | | |
| | | Interquartile Range | | |
| | | Skewness | | ,319 |
| | | Kurtosis | | ,628 |
| | lim_emocional | Mean | | 4,7051017 |
| | | 95% Confidence Interval for Mean | Lower Bound | |
| | | | Upper Bound | |
| | | 5% Trimmed Mean | | |
| | | Median | | |
| | | Variance | | |
| | | Std. Deviation | | |
| | | Minimum | | |
| | | Maximum | | |
| | | Range | | |
| | | Interquartile Range | | |
| | | Skewness | | ,319 |
| | | Kurtosis | | ,628 |
| Penetração | cap_func | Mean | | 6,745 |

**Descriptives**

| dispaurenia | | | | |
|---|---|---|---|---|
| | | 95% Confidence Interval for Mean | Lower Bound | |
| | | | Upper Bound | |
| | | 5% Trimmed Mean | | |
| | | Median | | |
| | | Variance | | |
| | | Std. Deviation | | |
| | | Minimum | | |
| | | Maximum | | |
| | | Range | | |
| | | Interquartile Range | | |
| | | Skewness | | |
| | | Kurtosis | | |
| | lim_emocional | Mean | | |
| | | 95% Confidence Interval for Mean | Lower Bound | |
| | | | Upper Bound | |
| | | 5% Trimmed Mean | | |
| | | Median | | |
| | | Variance | | |
| | | Std. Deviation | | |
| | | Minimum | | |
| | | Maximum | | |
| | | Range | | |
| | | Interquartile Range | | |
| | | Skewness | | |
| | | Kurtosis | | |
| Profundidade | cap_func | Mean | | |
| | | 95% Confidence Interval for Mean | Lower Bound | |
| | | | Upper Bound | |
| | | 5% Trimmed Mean | | |
| | | Median | | |
| | | Variance | | |
| | | Std. Deviation | | |
| | | Minimum | | |
| | | Maximum | | |
| | | Range | | |
| | | Interquartile Range | | |
| | | Skewness | | |
| | | Kurtosis | | |
| | lim_emocional | Mean | | |

**Descriptives**

| dispaurenia | | | | Statistic |
|---|---|---|---|---|
| | | 95% Confidence Interval for Mean | Lower Bound | 52,54 |
| | | | Upper Bound | 81,93 |
| | | 5% Trimmed Mean | | 68,87 |
| | | Median | | 75,00 |
| | | Variance | | 591,359 |
| | | Std. Deviation | | 24,318 |
| | | Minimum | | 10 |
| | | Maximum | | 95 |
| | | Range | | 85 |
| | | Interquartile Range | | 33 |
| | | Skewness | | -1,113 |
| | | Kurtosis | | 1,179 |
| | lim_emocional | Mean | | 41,051282 |
| | | 95% Confidence Interval for Mean | Lower Bound | 13,577763 |
| | | | Upper Bound | 68,524801 |
| | | 5% Trimmed Mean | | 40,056980 |
| | | Median | | 33,333333 |
| | | Variance | | 2066,960 |
| | | Std. Deviation | | 45,4638330 |
| | | Minimum | | ,0000 |
| | | Maximum | | 100,0000 |
| | | Range | | 100,0000 |
| | | Interquartile Range | | 100,0000 |
| | | Skewness | | ,441 |
| | | Kurtosis | | -1,804 |
| Profundidade | cap_func | Mean | | 81,33 |
| | | 95% Confidence Interval for Mean | Lower Bound | 73,69 |
| | | | Upper Bound | 88,97 |
| | | 5% Trimmed Mean | | 82,69 |
| | | Median | | 87,50 |
| | | Variance | | 327,449 |
| | | Std. Deviation | | 18,096 |
| | | Minimum | | 35 |
| | | Maximum | | 100 |
| | | Range | | 65 |
| | | Interquartile Range | | 31 |
| | | Skewness | | -,915 |
| | | Kurtosis | | ,035 |
| | lim_emocional | Mean | | 72,138889 |

**Descriptives**

| dispaurenia | | | | Std. Error |
|---|---|---|---|---|
| | | 95% Confidence Interval for Mean | Lower Bound | |
| | | | Upper Bound | |
| | | 5% Trimmed Mean | | |
| | | Median | | |
| | | Variance | | |
| | | Std. Deviation | | |
| | | Minimum | | |
| | | Maximum | | |
| | | Range | | |
| | | Interquartile Range | | |
| | | Skewness | | ,616 |
| | | Kurtosis | | 1,191 |
| | lim_emocional | Mean | | 12,6093986 |
| | | 95% Confidence Interval for Mean | Lower Bound | |
| | | | Upper Bound | |
| | | 5% Trimmed Mean | | |
| | | Median | | |
| | | Variance | | |
| | | Std. Deviation | | |
| | | Minimum | | |
| | | Maximum | | |
| | | Range | | |
| | | Interquartile Range | | |
| | | Skewness | | ,616 |
| | | Kurtosis | | 1,191 |
| Profundidade | cap_func | Mean | | 3,694 |
| | | 95% Confidence Interval for Mean | Lower Bound | |
| | | | Upper Bound | |
| | | 5% Trimmed Mean | | |
| | | Median | | |
| | | Variance | | |
| | | Std. Deviation | | |
| | | Minimum | | |
| | | Maximum | | |
| | | Range | | |
| | | Interquartile Range | | |
| | | Skewness | | ,472 |
| | | Kurtosis | | ,918 |
| | lim_emocional | Mean | | 8,4361034 |

**Descriptives**

| dispaurenia | | | |
|---|---|---|---|
| | | 95% Confidence Interval for Mean | Lower Bound |
| | | | Upper Bound |
| | | 5% Trimmed Mean | |
| | | Median | |
| | | Variance | |
| | | Std. Deviation | |
| | | Minimum | |
| | | Maximum | |
| | | Range | |
| | | Interquartile Range | |
| | | Skewness | |
| | | Kurtosis | |
| Penetraçao e Profundiddade | cap_func | Mean | |
| | | 95% Confidence Interval for Mean | Lower Bound |
| | | | Upper Bound |
| | | 5% Trimmed Mean | |
| | | Median | |
| | | Variance | |
| | | Std. Deviation | |
| | | Minimum | |
| | | Maximum | |
| | | Range | |
| | | Interquartile Range | |
| | | Skewness | |
| | | Kurtosis | |
| | lim_emocional | Mean | |
| | | 95% Confidence Interval for Mean | Lower Bound |
| | | | Upper Bound |
| | | 5% Trimmed Mean | |
| | | Median | |
| | | Variance | |
| | | Std. Deviation | |
| | | Minimum | |
| | | Maximum | |
| | | Range | |
| | | Interquartile Range | |
| | | Skewness | |
| | | Kurtosis | |

## Descriptives

| dispaurenia | | | | Statistic |
|---|---|---|---|---|
| | | 95% Confidence Interval for Mean | Lower Bound | 54,687479 |
| | | | Upper Bound | 89,590298 |
| | | 5% Trimmed Mean | | 74,598765 |
| | | Median | | 100,000000 |
| | | Variance | | 1708,028 |
| | | Std. Deviation | | 41,3282976 |
| | | Minimum | | ,0000 |
| | | Maximum | | 100,0000 |
| | | Range | | 100,0000 |
| | | Interquartile Range | | 58,7500 |
| | | Skewness | | -1,083 |
| | | Kurtosis | | -,595 |
| Penetraçao e Profundiddade | cap_func | Mean | | 81,92 |
| | | 95% Confidence Interval for Mean | Lower Bound | 73,07 |
| | | | Upper Bound | 90,78 |
| | | 5% Trimmed Mean | | 82,41 |
| | | Median | | 80,00 |
| | | Variance | | 214,744 |
| | | Std. Deviation | | 14,654 |
| | | Minimum | | 55 |
| | | Maximum | | 100 |
| | | Range | | 45 |
| | | Interquartile Range | | 28 |
| | | Skewness | | -,237 |
| | | Kurtosis | | -,925 |
| | lim_emocional | Mean | | 35,897436 |
| | | 95% Confidence Interval for Mean | Lower Bound | 10,602578 |
| | | | Upper Bound | 61,192294 |
| | | 5% Trimmed Mean | | 34,330484 |
| | | Median | | 33,333333 |
| | | Variance | | 1752,137 |
| | | Std. Deviation | | 41,8585326 |
| | | Minimum | | ,0000 |
| | | Maximum | | 100,0000 |
| | | Range | | 100,0000 |
| | | Interquartile Range | | 83,3333 |
| | | Skewness | | ,727 |
| | | Kurtosis | | -1,165 |

**Descriptives**

| dispaurenia | | | | Std. Error |
|---|---|---|---|---|
| | | 95% Confidence Interval for Mean | Lower Bound | |
| | | | Upper Bound | |
| | | 5% Trimmed Mean | | |
| | | Median | | |
| | | Variance | | |
| | | Std. Deviation | | |
| | | Minimum | | |
| | | Maximum | | |
| | | Range | | |
| | | Interquartile Range | | |
| | | Skewness | | ,472 |
| | | Kurtosis | | ,918 |
| Penetraçao e Profundiddade | cap_func | Mean | | 4,064 |
| | | 95% Confidence Interval for Mean | Lower Bound | |
| | | | Upper Bound | |
| | | 5% Trimmed Mean | | |
| | | Median | | |
| | | Variance | | |
| | | Std. Deviation | | |
| | | Minimum | | |
| | | Maximum | | |
| | | Range | | |
| | | Interquartile Range | | |
| | | Skewness | | ,616 |
| | | Kurtosis | | 1,191 |
| | lim_emocional | Mean | | 11,6094681 |
| | | 95% Confidence Interval for Mean | Lower Bound | |
| | | | Upper Bound | |
| | | 5% Trimmed Mean | | |
| | | Median | | |
| | | Variance | | |
| | | Std. Deviation | | |
| | | Minimum | | |
| | | Maximum | | |
| | | Range | | |
| | | Interquartile Range | | |
| | | Skewness | | ,616 |
| | | Kurtosis | | 1,191 |

```
 SPLIT FILE OFF.
*Nonparametric Tests: Independent Samples.
NPTESTS
  /INDEPENDENT TEST (cap_func lim_fisico dor lim_emocional) GROUP (dor_fora_menst) MANN_WHITNEY
  /MISSING SCOPE=ANALYSIS USERMISSING=EXCLUDE
  /CRITERIA ALPHA=0.05  CILEVEL=95.
```

# Nonparametric Tests

**Notes**

| | | |
|---|---|---|
| Output Created | | 08-MAY-2020 12:31:24 |
| Comments | | |
| Input | Data | \\Mac\iCloud\Lavoro\FMABC\Orientações\Orientações\finite\Marina Rodrigues - Caio e Fabia\Marina Farias\data_article_6.sav |
| | Active Dataset | InsiemeDati1 |
| | Filter | <none> |
| | Weight | <none> |
| | Split File | <none> |
| | N of Rows in Working Data File | 106 |
| Syntax | | NPTESTS /INDEPENDENT TEST (cap_func lim_fisico dor lim_emocional) GROUP (dor_fora_menst) MANN_WHITNEY /MISSING SCOPE=ANALYSIS USERMISSING=EXCLUDE /CRITERIA ALPHA=0.05 CILEVEL=95. |
| Resources | Processor Time | 00:00:00,08 |
| | Elapsed Time | 00:00:00,07 |

```
[InsiemeDati1] \\Mac\iCloud\Lavoro\FMABC\Orientações\Orientações\finite\Marin
a Rodrigues - Caio e Fabia\Marina Farias\data_article_6.sav
```

**Hypothesis Test Summary**

| | Null Hypothesis | Test | Sig. | Decision |
|---|---|---|---|---|
| 1 | The distribution of SF-36 - capacidade funcional is the same across categories of Dor fora da menstruaçao. | Independent -Samples Mann-Whitney U Test | ,207 | Retain the null hypothesis. |
| 2 | The distribution of SF-36 - limitacao fisica is the same across categories of Dor fora da menstruaçao. | Independent -Samples Mann-Whitney U Test | ,986 | Retain the null hypothesis. |
| 3 | The distribution of SF-36 - dor is the same across categories of Dor fora da menstruaçao. | Independent -Samples Mann-Whitney U Test | ,017 | Reject the null hypothesis. |
| 4 | The distribution of SF-36 - limites emocionais is the same across categories of Dor fora da menstruaçao. | Independent -Samples Mann-Whitney U Test | ,457 | Retain the null hypothesis. |

Asymptotic significances are displayed. The significance level is ,05.

```
 SORT CASES  BY dor_fora_menst.
SPLIT FILE LAYERED BY dor_fora_menst.
EXAMINE VARIABLES=dor
  /PLOT NONE
  /STATISTICS DESCRIPTIVES
  /CINTERVAL 95
  /MISSING LISTWISE
  /NOTOTAL.
```

# Explore

**Notes**

| | | |
|---|---|---|
| Output Created | | 08-MAY-2020 12:34:17 |
| Comments | | |
| Input | Data | \\Mac\iCloud\Lavoro\FMABC\Orientações\Orientações\finite\Marina Rodrigues - Caio e Fabia\Marina Farias\data_article_6.sav |
| | Active Dataset | InsiemeDati1 |
| | Filter | <none> |
| | Weight | <none> |
| | Split File | dor_fora_menst |
| | N of Rows in Working Data File | 106 |
| Missing Value Handling | Definition of Missing | User-defined missing values for dependent variables are treated as missing. |
| | Cases Used | Statistics are based on cases with no missing values for any dependent variable or factor used. |
| Syntax | | EXAMINE VARIABLES=dor<br>/PLOT NONE<br>/STATISTICS DESCRIPTIVES<br>/CINTERVAL 95<br>/MISSING LISTWISE<br>/NOTOTAL. |
| Resources | Processor Time | 00:00:00,00 |
| | Elapsed Time | 00:00:00,01 |

```
[InsiemeDati1] \\Mac\iCloud\Lavoro\FMABC\Orientações\Orientações\finite\Marin
a Rodrigues - Caio e Fabia\Marina Farias\data_article_6.sav
```

**Case Processing Summary**

| dor_fora_menst | | Cases | | | | | |
|---|---|---|---|---|---|---|---|
| | | Valid | | Missing | | Total | |
| | | N | Percent | N | Percent | N | Percent |
| não | dor | 85 | 100,0% | 0 | 0,0% | 85 | 100,0% |
| sim | dor | 21 | 100,0% | 0 | 0,0% | 21 | 100,0% |

**Descriptives**

| dor_fora_menst | | | | Statistic | Std. Error |
|---|---|---|---|---|---|
| não | dor | Mean | | 59,764706 | 2,7370089 |
| | | 95% Confidence Interval for Mean | Lower Bound | 54,321864 | |
| | | | Upper Bound | 65,207548 | |
| | | 5% Trimmed Mean | | 60,000000 | |
| | | Median | | 52,000000 | |
| | | Variance | | 636,754 | |
| | | Std. Deviation | | 25,2339751 | |
| | | Minimum | | 10,0000 | |
| | | Maximum | | 100,0000 | |
| | | Range | | 90,0000 | |
| | | Interquartile Range | | 43,0000 | |
| | | Skewness | | ,233 | ,261 |
| | | Kurtosis | | -1,000 | ,517 |
| sim | dor | Mean | | 73,428571 | 5,1480794 |
| | | 95% Confidence Interval for Mean | Lower Bound | 62,689866 | |
| | | | Upper Bound | 84,167277 | |
| | | 5% Trimmed Mean | | 74,891534 | |
| | | Median | | 84,000000 | |
| | | Variance | | 556,557 | |
| | | Std. Deviation | | 23,5914633 | |
| | | Minimum | | 20,0000 | |
| | | Maximum | | 100,0000 | |
| | | Range | | 80,0000 | |
| | | Interquartile Range | | 30,0000 | |
| | | Skewness | | -,750 | ,501 |
| | | Kurtosis | | -,157 | ,972 |

```
 SPLIT FILE OFF.
*Nonparametric Tests: Independent Samples.
NPTESTS
  /INDEPENDENT TEST (cap_func lim_fisico dor lim_emocional) GROUP (alt_intestinais) MANN_WHITNE
  /MISSING SCOPE=ANALYSIS USERMISSING=EXCLUDE
  /CRITERIA ALPHA=0.05  CILEVEL=95.
```

# Nonparametric Tests

**Notes**

| | | |
|---|---|---|
| Output Created | | 08-MAY-2020 12:35:43 |
| Comments | | |
| Input | Data | \\Mac\iCloud\Lavoro\FMABC\Orientações\Orientações\finite\Marina Rodrigues - Caio e Fabia\Marina Farias\data_article_6.sav |
| | Active Dataset | InsiemeDati1 |
| | Filter | <none> |
| | Weight | <none> |
| | Split File | <none> |
| | N of Rows in Working Data File | 106 |
| Syntax | | NPTESTS<br>/INDEPENDENT TEST (cap_func lim_fisico dor lim_emocional) GROUP (alt_intestinais) MANN_WHITNEY<br>/MISSING SCOPE=ANALYSIS USERMISSING=EXCLUDE<br>/CRITERIA ALPHA=0.05 CILEVEL=95. |
| Resources | Processor Time | 00:00:00,06 |
| | Elapsed Time | 00:00:00,06 |

```
[InsiemeDati1] \\Mac\iCloud\Lavoro\FMABC\Orientações\Orientações\finite\Marin
a Rodrigues - Caio e Fabia\Marina Farias\data_article_6.sav
```

**Hypothesis Test Summary**

| | Null Hypothesis | Test | Sig. | Decision |
|---|---|---|---|---|
| 1 | The distribution of SF-36 - capacidade funcional is the same across categories of alteraçao intestinal. | Independent-Samples Mann-Whitney U Test | ,218 | Retain the null hypothesis. |
| 2 | The distribution of SF-36 - limitacao fisica is the same across categories of alteraçao intestinal. | Independent-Samples Mann-Whitney U Test | ,626 | Retain the null hypothesis. |
| 3 | The distribution of SF-36 - dor is the same across categories of alteraçao intestinal. | Independent-Samples Mann-Whitney U Test | ,875 | Retain the null hypothesis. |
| 4 | The distribution of SF-36 - limites emocionais is the same across categories of alteraçao intestinal. | Independent-Samples Mann-Whitney U Test | ,461 | Retain the null hypothesis. |

Asymptotic significances are displayed. The significance level is ,05.

```
 *Nonparametric Tests: Independent Samples.
NPTESTS
  /INDEPENDENT TEST (cap_func lim_fisico dor lim_emocional) GROUP (alt_urinaria) MANN_WHITNEY
  /MISSING SCOPE=ANALYSIS USERMISSING=EXCLUDE
  /CRITERIA ALPHA=0.05  CILEVEL=95.
```

## Nonparametric Tests

**Notes**

| | | |
|---|---|---|
| Output Created | | 08-MAY-2020 12:35:58 |
| Comments | | |
| Input | Data | \\Mac\iCloud\Lavoro\FMABC\Orientações\Orientações\finite\Marina Rodrigues - Caio e Fabia\Marina Farias\data_article_6.sav |
| | Active Dataset | InsiemeDati1 |
| | Filter | <none> |
| | Weight | <none> |
| | Split File | <none> |
| | N of Rows in Working Data File | 106 |
| Syntax | | NPTESTS<br>/INDEPENDENT TEST (cap_func lim_fisico dor lim_emocional) GROUP (alt_urinaria) MANN_WHITNEY<br>/MISSING SCOPE=ANALYSIS USERMISSING=EXCLUDE<br>/CRITERIA ALPHA=0.05 CILEVEL=95. |
| Resources | Processor Time | 00:00:00,06 |
| | Elapsed Time | 00:00:00,08 |

```
[InsiemeDati1] \\Mac\iCloud\Lavoro\FMABC\Orientações\Orientações\finite\Marin
a Rodrigues - Caio e Fabia\Marina Farias\data_article_6.sav
```

### Hypothesis Test Summary

| | Null Hypothesis | Test | Sig. | Decision |
|---|---|---|---|---|
| 1 | The distribution of SF-36 - capacidade funcional is the same across categories of alteraçao urinaria. | Independent-Samples Mann-Whitney U Test | ,231 | Retain the null hypothesis. |
| 2 | The distribution of SF-36 - limitacao fisica is the same across categories of alteraçao urinaria. | Independent-Samples Mann-Whitney U Test | ,692 | Retain the null hypothesis. |
| 3 | The distribution of SF-36 - dor is the same across categories of alteraçao urinaria. | Independent-Samples Mann-Whitney U Test | ,412 | Retain the null hypothesis. |
| 4 | The distribution of SF-36 - limites emocionais is the same across categories of alteraçao urinaria. | Independent-Samples Mann-Whitney U Test | ,981 | Retain the null hypothesis. |

Asymptotic significances are displayed. The significance level is ,05.

```
FREQUENCIES VARIABLES=Grau_Endometriodo_Cat
  /STATISTICS=STDDEV MEAN
  /ORDER=ANALYSIS.
```

## Frequencies

**Notes**

| | | |
|---|---|---|
| Output Created | | 08-MAY-2020 18:44:07 |
| Comments | | |
| Input | Data | \\Mac\iCloud\Lavoro\FMABC\Orientações\Orientações\finite\Marina Rodrigues - Caio e Fabia\Marina Farias\data_article_6.sav |
| | Active Dataset | InsiemeDati1 |
| | Filter | <none> |
| | Weight | <none> |
| | Split File | <none> |
| | N of Rows in Working Data File | 106 |
| Missing Value Handling | Definition of Missing | User-defined missing values are treated as missing. |
| | Cases Used | Statistics are based on all cases with valid data. |
| Syntax | | FREQUENCIES VARIABLES=Grau_Endometriodo_Cat /STATISTICS=STDDEV MEAN /ORDER=ANALYSIS. |
| Resources | Processor Time | 00:00:00,00 |
| | Elapsed Time | 00:00:00,01 |

```
[InsiemeDati1] \\Mac\iCloud\Lavoro\FMABC\Orientações\Orientações\finite\Marina Rodrigues - Caio e Fabia\Marina Farias\data_article_6.sav
```

**Statistics**

Grau_Endometriodo_Cat

| | | |
|---|---|---|
| N | Valid | 106 |
| | Missing | 0 |
| Mean | | 1,75 |
| Std. Deviation | | ,432 |

**Grau_Endometriodo_Cat**

| | | Frequency | Percent | Valid Percent | Cumulative Percent |
|---|---|---|---|---|---|
| Valid | Grau I/II | 26 | 24,5 | 24,5 | 24,5 |
| | Grau III/IV | 80 | 75,5 | 75,5 | 100,0 |
| | Total | 106 | 100,0 | 100,0 | |
